# Supplementary material for: The Emerging Role of PET/CT with PSMA-Targeting Radiopharmaceuticals in Clear Cell Renal Cancer: An Updated Systematic Review
Source: Cancers (Basel). 2023 Jan 5;15(2):355. doi: 10.3390/cancers15020355 (PMC9857064; doi:10.3390/cancers15020355)
Supplement: Supplementary file 1 [file cancers-15-00355-s001.zip › Supplementary table S2.docx]

| **Excluded studies** | **Reason of exclusion** |
| --- | --- |
| Foss, C.A.; Mease, R.C.; Fan, H.; Wang, Y.; Ravert, H.T.; Dannals, R.F.; Olszewski, R.T.; Heston, W.D.; Kozikowski, A.P.; Pomper, M.G. Radiolabeled Small-Molecule Ligands for Prostate-Specific Membrane Antigen: In Vivo Imaging in Experimental Models of Prostate Cancer. *Clin Cancer Res* **2005**, *11*, 4022–4028, doi:[10.1158/1078-0432.CCR-04-2690](https://doi.org/10.1158/1078-0432.CCR-04-2690). | Original study not in the field of interest |
| Chopra, A. N-[N-[(S)-1,3-Dicarboxypropyl]Carbamoyl]-S-[11C]Methyl-L-Cysteine. In *Molecular Imaging and Contrast Agent Database (MICAD)*; National Center for Biotechnology Information (US): Bethesda (MD), **2004**. | Book chapter not in the field of interest |
| Mease, R.C.; Dusich, C.L.; Foss, C.A.; Ravert, H.T.; Dannals, R.F.; Seidel, J.; Prideaux, A.; Fox, J.J.; Sgouros, G.; Kozikowski, A.P.; et al. N-[N-[(S)-1,3-Dicarboxypropyl]Carbamoyl]-4-[18F]Fluorobenzyl-L-Cysteine, [18F]DCFBC: A New Imaging Probe for Prostate Cancer. *Clin Cancer Res* **2008**, *14*, 3036–3043, doi:[10.1158/1078-0432.CCR-07-1517](https://doi.org/10.1158/1078-0432.CCR-07-1517). | Original study not in the field of interest |
| Banerjee, S.R.; Foss, C.A.; Castanares, M.; Mease, R.C.; Byun, Y.; Fox, J.J.; Hilton, J.; Lupold, S.E.; Kozikowski, A.P.; Pomper, M.G. Synthesis and Evaluation of Technetium-99m- and Rhenium-Labeled Inhibitors of the Prostate-Specific Membrane Antigen (PSMA). *J Med Chem* **2008**, *51*, 4504–4517, doi:[10.1021/jm800111u](https://doi.org/10.1021/jm800111u). | Original study not in the field of interest |
| Leung, K. N-[N-[(S)-1,3-Dicarboxypropyl]Carbamoyl]-4-[18F]Fluorobenzyl-L-Cysteine. In *Molecular Imaging and Contrast Agent Database (MICAD)*; National Center for Biotechnology Information (US): Bethesda (MD), **2004**. | Book chapter not in the field of interest |
| Leung, K. 64Cu-1,4,7,10-Tetraazacyclododecane-N,N’,N’’,N’’’-Tetraacetic Acid-Anti-Prostate-Specific Membrane Antigen 3/A12 Monoclonal Antibody. In *Molecular Imaging and Contrast Agent Database (MICAD)*; National Center for Biotechnology Information (US): Bethesda (MD), **2004**. | Book chapter not in the field of interest |
| Lapi, S.E.; Wahnishe, H.; Pham, D.; Wu, L.Y.; Nedrow-Byers, J.R.; Liu, T.; Vejdani, K.; VanBrocklin, H.F.; Berkman, C.E.; Jones, E.F. Assessment of an 18F-Labeled Phosphoramidate Peptidomimetic as a New Prostate-Specific Membrane Antigen-Targeted Imaging Agent for Prostate Cancer. *J Nucl Med* **2009**, *50*, 2042–2048, doi:[10.2967/jnumed.109.066589](https://doi.org/10.2967/jnumed.109.066589). | Original study not in the field of interest |
| Kim, D.; Jeong, Y.Y.; Jon, S. A Drug-Loaded Aptamer-Gold Nanoparticle Bioconjugate for Combined CT Imaging and Therapy of Prostate Cancer. *ACS Nano* **2010**, *4*, 3689–3696, doi:[10.1021/nn901877h](https://doi.org/10.1021/nn901877h). | Original study not in the field of interest |
| Alt, K.; Wiehr, S.; Ehrlichmann, W.; Reischl, G.; Wolf, P.; Pichler, B.J.; Elsässer-Beile, U.; Bühler, P. High-Resolution Animal PET Imaging of Prostate Cancer Xenografts with Three Different 64Cu-Labeled Antibodies against Native Cell-Adherent PSMA. *Prostate* **2010**, *70*, 1413–1421, doi:[10.1002/pros.21176](https://doi.org/10.1002/pros.21176). | Original study not in the field of interest |
| Leung, K. 89Zr-Desferrioxamine B-J591 Anti-Prostate-Specific Membrane Antigen Monoclonal Antibody. In *Molecular Imaging and Contrast Agent Database (MICAD)*; National Center for Biotechnology Information (US): Bethesda (MD), **2004**. | Book chapter not in the field of interest |
| Leung, K. 89Zr-Desferrioxamine B-7E11 Anti-Prostate-Specific Membrane Antigen Monoclonal Antibody. In *Molecular Imaging and Contrast Agent Database (MICAD)*; National Center for Biotechnology Information (US): Bethesda (MD), **2004**. | Book chapter not in the field of interest |
| Osborne, J.R.; Akhtar, N.H.; Vallabhajosula, S.; Anand, A.; Deh, K.; Tagawa, S.T. Prostate-Specific Membrane Antigen-Based Imaging. *Urol Oncol* **2013**, *31*, 144–154, doi:[10.1016/j.urolonc.2012.04.016](https://doi.org/10.1016/j.urolonc.2012.04.016). | Review not in the field of interest |
| Afshar-Oromieh, A.; Malcher, A.; Eder, M.; Eisenhut, M.; Linhart, H.G.; Hadaschik, B.A.; Holland-Letz, T.; Giesel, F.L.; Kratochwil, C.; Haufe, S.; et al. PET Imaging with a [68Ga]Gallium-Labelled PSMA Ligand for the Diagnosis of Prostate Cancer: Biodistribution in Humans and First Evaluation of Tumour Lesions. *Eur J Nucl Med Mol Imaging* **2013**, *40*, 486–495, doi:[10.1007/s00259-012-2298-2](https://doi.org/10.1007/s00259-012-2298-2). | Original study not in the field of interest |
| Hao, G.; Kumar, A.; Dobin, T.; Oz, O.K.; Hsieh, J.-T.; Sun, X. A Multivalent Approach of Imaging Probe Design to Overcome an Endogenous Anion Binding Competition for Noninvasive Assessment of Prostate Specific Membrane Antigen. *Mol Pharm* **2013**, *10*, 2975–2985, doi:[10.1021/mp4000844](https://doi.org/10.1021/mp4000844). | Original study not in the field of interest |
| Lesche, R.; Kettschau, G.; Gromov, A.V.; Böhnke, N.; Borkowski, S.; Mönning, U.; Hegele-Hartung, C.; Döhr, O.; Dinkelborg, L.M.; Graham, K. Preclinical Evaluation of BAY 1075553, a Novel (18)F-Labelled Inhibitor of Prostate-Specific Membrane Antigen for PET Imaging of Prostate Cancer. *Eur J Nucl Med Mol Imaging* **2014**, *41*, 89–101, doi:[10.1007/s00259-013-2527-3](https://doi.org/10.1007/s00259-013-2527-3). | Original study not in the field of interest |
| Afshar-Oromieh, A.; Haberkorn, U.; Schlemmer, H.P.; Fenchel, M.; Eder, M.; Eisenhut, M.; Hadaschik, B.A.; Kopp-Schneider, A.; Röthke, M. Comparison of PET/CT and PET/MRI Hybrid Systems Using a 68Ga-Labelled PSMA Ligand for the Diagnosis of Recurrent Prostate Cancer: Initial Experience. *Eur J Nucl Med Mol Imaging* **2014**, *41*, 887–897, doi:[10.1007/s00259-013-2660-z](https://doi.org/10.1007/s00259-013-2660-z). | Original study not in the field of interest |
| Zechmann, C.M.; Afshar-Oromieh, A.; Armor, T.; Stubbs, J.B.; Mier, W.; Hadaschik, B.; Joyal, J.; Kopka, K.; Debus, J.; Babich, J.W.; et al. Radiation Dosimetry and First Therapy Results with a (124)I/ (131)I-Labeled Small Molecule (MIP-1095) Targeting PSMA for Prostate Cancer Therapy. *Eur J Nucl Med Mol Imaging* **2014**, *41*, 1280–1292, doi:[10.1007/s00259-014-2713-y](https://doi.org/10.1007/s00259-014-2713-y). | Original study not in the field of interest |
| Wiehr, S.; Bühler, P.; Gierschner, D.; Wolf, P.; Rolle, A.-M.; Kesenheimer, C.; Pichler, B.J.; Elsässer-Beile, U. Pharmacokinetics and PET Imaging Properties of Two Recombinant Anti-PSMA Antibody Fragments in Comparison to Their Parental Antibody. *Prostate* **2014**, *74*, 743–755, doi:[10.1002/pros.22794](https://doi.org/10.1002/pros.22794). | Original study not in the field of interest |
| Huang, S.S.; Wang, X.; Zhang, Y.; Doke, A.; DiFilippo, F.P.; Heston, W.D. Improving the Biodistribution of PSMA-Targeting Tracers with a Highly Negatively Charged Linker. *Prostate* **2014**, *74*, 702–713, doi:[10.1002/pros.22789](https://doi.org/10.1002/pros.22789). | Original study not in the field of interest |
| Tolmachev, V.; Malmberg, J.; Estrada, S.; Eriksson, O.; Orlova, A. Development of a 124I-Labeled Version of the Anti-PSMA Monoclonal Antibody Capromab for ImmunoPET Staging of Prostate Cancer: Aspects of Labeling Chemistry and Biodistribution. *Int J Oncol* **2014**, *44*, 1998–2008, doi:[10.3892/ijo.2014.2376](https://doi.org/10.3892/ijo.2014.2376). | Original study not in the field of interest |
| Demirci, E.; Ocak, M.; Kabasakal, L.; Decristoforo, C.; Talat, Z.; Halaç, M.; Kanmaz, B. (68)Ga-PSMA PET/CT Imaging of Metastatic Clear Cell Renal Cell Carcinoma. *Eur J Nucl Med Mol Imaging* **2014**, *41*, 1461–1462, doi:[10.1007/s00259-014-2766-y](https://doi.org/10.1007/s00259-014-2766-y). | Case report in the field of interest |
| Banerjee, S.R.; Foss, C.A.; Pullambhatla, M.; Wang, Y.; Srinivasan, S.; Hobbs, R.F.; Baidoo, K.E.; Brechbiel, M.W.; Nimmagadda, S.; Mease, R.C.; et al. Preclinical Evaluation of 86Y-Labeled Inhibitors of Prostate-Specific Membrane Antigen for Dosimetry Estimates. *J Nucl Med* **2015**, *56*, 628–634, doi:[10.2967/jnumed.114.149062](https://doi.org/10.2967/jnumed.114.149062). | Original study not in the field of interest |
| Benešová, M.; Schäfer, M.; Bauder-Wüst, U.; Afshar-Oromieh, A.; Kratochwil, C.; Mier, W.; Haberkorn, U.; Kopka, K.; Eder, M. Preclinical Evaluation of a Tailor-Made DOTA-Conjugated PSMA Inhibitor with Optimized Linker Moiety for Imaging and Endoradiotherapy of Prostate Cancer. *J Nucl Med* **2015**, *56*, 914–920, doi:[10.2967/jnumed.114.147413](https://doi.org/10.2967/jnumed.114.147413). | Original study not in the field of interest |
| Herrmann, K.; Bluemel, C.; Weineisen, M.; Schottelius, M.; Wester, H.-J.; Czernin, J.; Eberlein, U.; Beykan, S.; Lapa, C.; Riedmiller, H.; et al. Biodistribution and Radiation Dosimetry for a Probe Targeting Prostate-Specific Membrane Antigen for Imaging and Therapy. *J Nucl Med* **2015**, *56*, 855–861, doi:[10.2967/jnumed.115.156133](https://doi.org/10.2967/jnumed.115.156133). | Original study not in the field of interest |
| Dietlein, M.; Kobe, C.; Kuhnert, G.; Stockter, S.; Fischer, T.; Schomäcker, K.; Schmidt, M.; Dietlein, F.; Zlatopolskiy, B.D.; Krapf, P.; et al. Comparison of [(18)F]DCFPyL and [ (68)Ga]Ga-PSMA-HBED-CC for PSMA-PET Imaging in Patients with Relapsed Prostate Cancer. *Mol Imaging Biol* **2015**, *17*, 575–584, doi:[10.1007/s11307-015-0866-0](https://doi.org/10.1007/s11307-015-0866-0). | Original study not in the field of interest |
| Weineisen, M.; Schottelius, M.; Simecek, J.; Baum, R.P.; Yildiz, A.; Beykan, S.; Kulkarni, H.R.; Lassmann, M.; Klette, I.; Eiber, M.; et al. 68Ga- and 177Lu-Labeled PSMA I&T: Optimization of a PSMA-Targeted Theranostic Concept and First Proof-of-Concept Human Studies. *J Nucl Med* **2015**, *56*, 1169–1176, doi:[10.2967/jnumed.115.158550](https://doi.org/10.2967/jnumed.115.158550). | Original study not in the field of interest |
| Ahmadzadehfar, H.; Rahbar, K.; Kürpig, S.; Bögemann, M.; Claesener, M.; Eppard, E.; Gärtner, F.; Rogenhofer, S.; Schäfers, M.; Essler, M. Early Side Effects and First Results of Radioligand Therapy with (177)Lu-DKFZ-617 PSMA of Castrate-Resistant Metastatic Prostate Cancer: A Two-Centre Study. *EJNMMI Res* **2015**, *5*, 114, doi:[10.1186/s13550-015-0114-2](https://doi.org/10.1186/s13550-015-0114-2). | Original study not in the field of interest |
| Maurer, T.; Kübler, H.; Gschwend, J.E.; Eiber, M. [Positron-emission tomography in urooncology]. *Urologe A* **2015**, *54*, 983–991, doi:[10.1007/s00120-015-3868-2](https://doi.org/10.1007/s00120-015-3868-2). | Review not in the field of interest |
| Fendler, W.P.; Wenter, V.; Stief, C.G.; Gratzke, C.; Bartenstein, P. [Radionuclide therapy and diagnostics in urology]. *Urologe A* **2015**, *54*, 1025–1035; quiz 1036–1037, doi:[10.1007/s00120-014-3730-y](https://doi.org/10.1007/s00120-014-3730-y). | Review not in the field of interest |
| Kabasakal, L.; AbuQbeitah, M.; Aygün, A.; Yeyin, N.; Ocak, M.; Demirci, E.; Toklu, T. Pre-Therapeutic Dosimetry of Normal Organs and Tissues of (177)Lu-PSMA-617 Prostate-Specific Membrane Antigen (PSMA) Inhibitor in Patients with Castration-Resistant Prostate Cancer. *Eur J Nucl Med Mol Imaging* **2015**, *42*, 1976–1983, doi:[10.1007/s00259-015-3125-3](https://doi.org/10.1007/s00259-015-3125-3). | Original study not in the field of interest |
| Rowe, S.P.; Gorin, M.A.; Hammers, H.J.; Som Javadi, M.; Hawasli, H.; Szabo, Z.; Cho, S.Y.; Pomper, M.G.; Allaf, M.E. Imaging of Metastatic Clear Cell Renal Cell Carcinoma with PSMA-Targeted ^18^F-DCFPyL PET/CT. *Ann Nucl Med* **2015**, *29*, 877–882, doi:[10.1007/s12149-015-1017-z](https://doi.org/10.1007/s12149-015-1017-z). | Small case series in the field of interest |
| Sawicki, L.M.; Buchbender, C.; Boos, J.; Giessing, M.; Ermert, J.; Antke, C.; Antoch, G.; Hautzel, H. Diagnostic Potential of PET/CT Using a 68Ga-Labelled Prostate-Specific Membrane Antigen Ligand in Whole-Body Staging of Renal Cell Carcinoma: Initial Experience. *Eur J Nucl Med Mol Imaging* **2017**, *44*, 102–107, doi:[10.1007/s00259-016-3360-2](https://doi.org/10.1007/s00259-016-3360-2). | Small case series in the field of interest |
| Siva, S.; Callahan, J.; Pryor, D.; Martin, J.; Lawrentschuk, N.; Hofman, M.S. Utility of 68 Ga Prostate Specific Membrane Antigen - Positron Emission Tomography in Diagnosis and Response Assessment of Recurrent Renal Cell Carcinoma. *J Med Imaging Radiat Oncol* **2017**, *61*, 372–378, doi:[10.1111/1754-9485.12590](https://doi.org/10.1111/1754-9485.12590). | Small case series in the field of interest |
| Afshar-Oromieh, A.; Hetzheim, H.; Kratochwil, C.; Benesova, M.; Eder, M.; Neels, O.C.; Eisenhut, M.; Kübler, W.; Holland-Letz, T.; Giesel, F.L.; et al. The Theranostic PSMA Ligand PSMA-617 in the Diagnosis of Prostate Cancer by PET/CT: Biodistribution in Humans, Radiation Dosimetry, and First Evaluation of Tumor Lesions. *J Nucl Med* **2015**, *56*, 1697–1705, doi:[10.2967/jnumed.115.161299](https://doi.org/10.2967/jnumed.115.161299). | Original study not in the field of interest |
| Rowe, S.P.; Gorin, M.A.; Hammers, H.J.; Pomper, M.G.; Allaf, M.E.; Javadi, M.S. Detection of 18F-FDG PET/CT Occult Lesions With 18F-DCFPyL PET/CT in a Patient With Metastatic Renal Cell Carcinoma. *Clin Nucl Med* **2016**, *41*, 83–85, doi:[10.1097/RLU.0000000000000995](https://doi.org/10.1097/RLU.0000000000000995). | Case report in the field of interest |
| Nedrow, J.R.; Latoche, J.D.; Day, K.E.; Modi, J.; Ganguly, T.; Zeng, D.; Kurland, B.F.; Berkman, C.E.; Anderson, C.J. Targeting PSMA with a Cu-64 Labeled Phosphoramidate Inhibitor for PET/CT Imaging of Variant PSMA-Expressing Xenografts in Mouse Models of Prostate Cancer. *Mol Imaging Biol* **2016**, *18*, 402–410, doi:[10.1007/s11307-015-0908-7](https://doi.org/10.1007/s11307-015-0908-7). | Original study not in the field of interest |
| Fan, W.; Zhang, Z.; Zhu, Z.; Yang, D.; Chen, X.; Wang, J.; Chen, F.; Song, X. Synthesis and Positron Emission Tomography Evaluation of 18F-Glu-Urea-Lys, a Prostate-Specific Membrane Antigen-Based Imaging Agent for Prostate Cancer. *Oncol Lett* **2015**, *10*, 2299–2302, doi:[10.3892/ol.2015.3625](https://doi.org/10.3892/ol.2015.3625). | Original study not in the field of interest |
| Yang, X.; Mease, R.C.; Pullambhatla, M.; Lisok, A.; Chen, Y.; Foss, C.A.; Wang, Y.; Shallal, H.; Edelman, H.; Hoye, A.T.; et al. [(18)F]Fluorobenzoyllysinepentanedioic Acid Carbamates: New Scaffolds for Positron Emission Tomography (PET) Imaging of Prostate-Specific Membrane Antigen (PSMA). *J Med Chem* **2016**, *59*, 206–218, doi:[10.1021/acs.jmedchem.5b01268](https://doi.org/10.1021/acs.jmedchem.5b01268). | Original study not in the field of interest |
| Minamimoto, R.; Hancock, S.; Schneider, B.; Chin, F.T.; Jamali, M.; Loening, A.; Vasanawala, S.; Gambhir, S.S.; Iagaru, A. Pilot Comparison of ^68^Ga-RM2 PET and ^68^Ga-PSMA-11 PET in Patients with Biochemically Recurrent Prostate Cancer. *J Nucl Med* **2016**, *57*, 557–562, doi:[10.2967/jnumed.115.168393](https://doi.org/10.2967/jnumed.115.168393). | Original study not in the field of interest |
| Gourni, E.; Canovas, C.; Goncalves, V.; Denat, F.; Meyer, P.T.; Maecke, H.R. (R)-NODAGA-PSMA: A Versatile Precursor for Radiometal Labeling and Nuclear Imaging of PSMA-Positive Tumors. *PLoS One* **2015**, *10*, e0145755, doi:[10.1371/journal.pone.0145755](https://doi.org/10.1371/journal.pone.0145755). | Original study not in the field of interest |
| Liolios, C.; Schäfer, M.; Haberkorn, U.; Eder, M.; Kopka, K. Novel Bispecific PSMA/GRPr Targeting Radioligands with Optimized Pharmacokinetics for Improved PET Imaging of Prostate Cancer. *Bioconjug Chem* **2016**, *27*, 737–751, doi:[10.1021/acs.bioconjchem.5b00687](https://doi.org/10.1021/acs.bioconjchem.5b00687). | Original study not in the field of interest |
| Baum, R.P.; Kulkarni, H.R.; Schuchardt, C.; Singh, A.; Wirtz, M.; Wiessalla, S.; Schottelius, M.; Mueller, D.; Klette, I.; Wester, H.-J. 177Lu-Labeled Prostate-Specific Membrane Antigen Radioligand Therapy of Metastatic Castration-Resistant Prostate Cancer: Safety and Efficacy. *J Nucl Med* **2016**, *57*, 1006–1013, doi:[10.2967/jnumed.115.168443](https://doi.org/10.2967/jnumed.115.168443). | Original study not in the field of interest |
| Einspieler, I.; Tauber, R.; Maurer, T.; Schwaiger, M.; Eiber, M. 68Ga Prostate-Specific Membrane Antigen Uptake in Renal Cell Cancer Lymph Node Metastases. *Clin Nucl Med* **2016**, *41*, e261-262, doi:[10.1097/RLU.0000000000001128](https://doi.org/10.1097/RLU.0000000000001128). | Case report in the field of interest |
| Prasad, V.; Steffen, I.G.; Diederichs, G.; Makowski, M.R.; Wust, P.; Brenner, W. Biodistribution of [(68)Ga]PSMA-HBED-CC in Patients with Prostate Cancer: Characterization of Uptake in Normal Organs and Tumour Lesions. *Mol Imaging Biol* **2016**, *18*, 428–436, doi:[10.1007/s11307-016-0945-x](https://doi.org/10.1007/s11307-016-0945-x). | Original study not in the field of interest |
| Rahbar, K.; Schmidt, M.; Heinzel, A.; Eppard, E.; Bode, A.; Yordanova, A.; Claesener, M.; Ahmadzadehfar, H. Response and Tolerability of a Single Dose of 177Lu-PSMA-617 in Patients with Metastatic Castration-Resistant Prostate Cancer: A Multicenter Retrospective Analysis. *J Nucl Med* **2016**, *57*, 1334–1338, doi:[10.2967/jnumed.116.173757](https://doi.org/10.2967/jnumed.116.173757). | Original study not in the field of interest |
| Ray Banerjee, S.; Chen, Z.; Pullambhatla, M.; Lisok, A.; Chen, J.; Mease, R.C.; Pomper, M.G. Preclinical Comparative Study of (68)Ga-Labeled DOTA, NOTA, and HBED-CC Chelated Radiotracers for Targeting PSMA. *Bioconjug Chem* **2016**, *27*, 1447–1455, doi:[10.1021/acs.bioconjchem.5b00679](https://doi.org/10.1021/acs.bioconjchem.5b00679). | Original study not in the field of interest |
| Das, T.; Guleria, M.; Parab, A.; Kale, C.; Shah, H.; Sarma, H.D.; Lele, V.R.; Banerjee, S. Clinical Translation of (177)Lu-Labeled PSMA-617: Initial Experience in Prostate Cancer Patients. *Nucl Med Biol* **2016**, *43*, 296–302, doi:[10.1016/j.nucmedbio.2016.02.002](https://doi.org/10.1016/j.nucmedbio.2016.02.002). | Original study not in the field of interest |
| Pfob, C.H.; Ziegler, S.; Graner, F.P.; Köhner, M.; Schachoff, S.; Blechert, B.; Wester, H.-J.; Scheidhauer, K.; Schwaiger, M.; Maurer, T.; et al. Biodistribution and Radiation Dosimetry of (68)Ga-PSMA HBED CC-a PSMA Specific Probe for PET Imaging of Prostate Cancer. *Eur J Nucl Med Mol Imaging* **2016**, *43*, 1962–1970, doi:[10.1007/s00259-016-3424-3](https://doi.org/10.1007/s00259-016-3424-3). | Case series not in the field of interest |
| Afshar-Oromieh, A.; Hetzheim, H.; Kübler, W.; Kratochwil, C.; Giesel, F.L.; Hope, T.A.; Eder, M.; Eisenhut, M.; Kopka, K.; Haberkorn, U. Radiation Dosimetry of (68)Ga-PSMA-11 (HBED-CC) and Preliminary Evaluation of Optimal Imaging Timing. *Eur J Nucl Med Mol Imaging* **2016**, *43*, 1611–1620, doi:[10.1007/s00259-016-3419-0](https://doi.org/10.1007/s00259-016-3419-0). | Original study not in the field of interest |
| Chen, Y.; Lisok, A.; Chatterjee, S.; Wharram, B.; Pullambhatla, M.; Wang, Y.; Sgouros, G.; Mease, R.C.; Pomper, M.G. [(18)F]Fluoroethyl Triazole Substituted PSMA Inhibitor Exhibiting Rapid Normal Organ Clearance. *Bioconjug Chem* **2016**, *27*, 1655–1662, doi:[10.1021/acs.bioconjchem.6b00195](https://doi.org/10.1021/acs.bioconjchem.6b00195). | Original study not in the field of interest |
| Wüstemann, T.; Bauder-Wüst, U.; Schäfer, M.; Eder, M.; Benesova, M.; Leotta, K.; Kratochwil, C.; Haberkorn, U.; Kopka, K.; Mier, W. Design of Internalizing PSMA-Specific Glu-Ureido-Based Radiotherapeuticals. *Theranostics* **2016**, *6*, 1085–1095, doi:[10.7150/thno.13448](https://doi.org/10.7150/thno.13448). | Original study not in the field of interest |
| Boschi, S.; Lee, J.T.; Beykan, S.; Slavik, R.; Wei, L.; Spick, C.; Eberlein, U.; Buck, A.K.; Lodi, F.; Cicoria, G.; et al. Synthesis and Preclinical Evaluation of an Al18F Radiofluorinated GLU-UREA-LYS(AHX)-HBED-CC PSMA Ligand. *Eur J Nucl Med Mol Imaging* **2016**, *43*, 2122–2130, doi:[10.1007/s00259-016-3437-y](https://doi.org/10.1007/s00259-016-3437-y). | Original study not in the field of interest |
| Demirci, E.; Sahin, O.E.; Ocak, M.; Akovali, B.; Nematyazar, J.; Kabasakal, L. Normal Distribution Pattern and Physiological Variants of 68Ga-PSMA-11 PET/CT Imaging. *Nucl Med Commun* **2016**, *37*, 1169–1179, doi:[10.1097/MNM.0000000000000566](https://doi.org/10.1097/MNM.0000000000000566). | Original study not in the field of interest |
| Gorin, M.A.; Rowe, S.P.; Hooper, J.E.; Kates, M.; Hammers, H.-J.; Szabo, Z.; Pomper, M.G.; Allaf, M.E. PSMA-Targeted 18F-DCFPyL PET/CT Imaging of Clear Cell Renal Cell Carcinoma: Results from a Rapid Autopsy. *Eur Urol* **2017**, *71*, 145–146, doi:[10.1016/j.eururo.2016.06.019](https://doi.org/10.1016/j.eururo.2016.06.019). | Case report in the field of interest |
| Gupta, M.; Choudhury, P.S.; Gupta, G.; Gandhi, J. Metastasis in Urothelial Carcinoma Mimicking Prostate Cancer Metastasis in Ga-68 Prostate-Specific Membrane Antigen Positron Emission Tomography-Computed Tomography in a Case of Synchronous Malignancy. *Indian J Nucl Med* **2016**, *31*, 222–224, doi:[10.4103/0972-3919.183615](https://doi.org/10.4103/0972-3919.183615). | Case report not in the field of interest |
| Pandit-Taskar, N.; O’Donoghue, J.A.; Ruan, S.; Lyashchenko, S.K.; Carrasquillo, J.A.; Heller, G.; Martinez, D.F.; Cheal, S.M.; Lewis, J.S.; Fleisher, M.; et al. First-in-Human Imaging with 89Zr-Df-IAB2M Anti-PSMA Minibody in Patients with Metastatic Prostate Cancer: Pharmacokinetics, Biodistribution, Dosimetry, and Lesion Uptake. *J Nucl Med* **2016**, *57*, 1858–1864, doi:[10.2967/jnumed.116.176206](https://doi.org/10.2967/jnumed.116.176206). | Original study not in the field of interest |
| D’Souza, J.W.; Hensley, H.; Doss, M.; Beigarten, C.; Torgov, M.; Olafsen, T.; Yu, J.Q.; Robinson, M.K. Cerenkov Luminescence Imaging as a Modality to Evaluate Antibody-Based PET Radiotracers. *J Nucl Med* **2017**, *58*, 175–180, doi:[10.2967/jnumed.116.178780](https://doi.org/10.2967/jnumed.116.178780). | Original study not in the field of interest |
| Rhee, H.; Ng, K.L.; Tse, B.W.-C.; Yeh, M.-C.; Russell, P.J.; Nelson, C.; Thomas, P.; Samaratunga, H.; Vela, I.; Gobe, G.; et al. Using Prostate Specific Membrane Antigen (PSMA) Expression in Clear Cell Renal Cell Carcinoma for Imaging Advanced Disease. *Pathology* **2016**, *48*, 613–616, doi:[10.1016/j.pathol.2016.05.011](https://doi.org/10.1016/j.pathol.2016.05.011). | Case report in the field of interest |
| Ferdinandus, J.; Eppard, E.; Gaertner, F.C.; Kürpig, S.; Fimmers, R.; Yordanova, A.; Hauser, S.; Feldmann, G.; Essler, M.; Ahmadzadehfar, H. Predictors of Response to Radioligand Therapy of Metastatic Castrate-Resistant Prostate Cancer with 177Lu-PSMA-617. *J Nucl Med* **2017**, *58*, 312–319, doi:[10.2967/jnumed.116.178228](https://doi.org/10.2967/jnumed.116.178228). | Original study not in the field of interest |
| Kletting, P.; Schuchardt, C.; Kulkarni, H.R.; Shahinfar, M.; Singh, A.; Glatting, G.; Baum, R.P.; Beer, A.J. Investigating the Effect of Ligand Amount and Injected Therapeutic Activity: A Simulation Study for 177Lu-Labeled PSMA-Targeting Peptides. *PLoS One* **2016**, *11*, e0162303, doi:[10.1371/journal.pone.0162303](https://doi.org/10.1371/journal.pone.0162303). | Original study not in the field of interest |
| Okamoto, S.; Thieme, A.; Allmann, J.; D’Alessandria, C.; Maurer, T.; Retz, M.; Tauber, R.; Heck, M.M.; Wester, H.-J.; Tamaki, N.; et al. Radiation Dosimetry for 177Lu-PSMA I&T in Metastatic Castration-Resistant Prostate Cancer: Absorbed Dose in Normal Organs and Tumor Lesions. *J Nucl Med* **2017**, *58*, 445–450, doi:[10.2967/jnumed.116.178483](https://doi.org/10.2967/jnumed.116.178483). | Original study not in the field of interest |
| Fendler, W.P.; Reinhardt, S.; Ilhan, H.; Delker, A.; Böning, G.; Gildehaus, F.J.; Stief, C.; Bartenstein, P.; Gratzke, C.; Lehner, S.; et al. Preliminary Experience with Dosimetry, Response and Patient Reported Outcome after 177Lu-PSMA-617 Therapy for Metastatic Castration-Resistant Prostate Cancer. *Oncotarget* **2017**, *8*, 3581–3590, doi:[10.18632/oncotarget.12240](https://doi.org/10.18632/oncotarget.12240). | Original study not in the field of interest |
| Grubmüller, B.; Baum, R.P.; Capasso, E.; Singh, A.; Ahmadi, Y.; Knoll, P.; Floth, A.; Righi, S.; Zandieh, S.; Meleddu, C.; et al. 64Cu-PSMA-617 PET/CT Imaging of Prostate Adenocarcinoma: First In-Human Studies. *Cancer Biother Radiopharm* **2016**, *31*, 277–286, doi:[10.1089/cbr.2015.1964](https://doi.org/10.1089/cbr.2015.1964). | Original study not in the field of interest |
| Sasikumar, A.; Joy, A.; Nanabala, R.; Unni, M.; Tk, P. Complimentary Pattern of Uptake in 18F-FDG PET/CT and 68Ga-Prostate-Specific Membrane Antigen PET/CT in a Case of Metastatic Clear Cell Renal Carcinoma. *Clin Nucl Med* **2016**, *41*, e517–e519, doi:[10.1097/RLU.0000000000001394](https://doi.org/10.1097/RLU.0000000000001394). | Case report in the field of interest |
| Yadav, M.P.; Ballal, S.; Tripathi, M.; Damle, N.A.; Sahoo, R.K.; Seth, A.; Bal, C. Post-Therapeutic Dosimetry of 177Lu-DKFZ-PSMA-617 in the Treatment of Patients with Metastatic Castration-Resistant Prostate Cancer. *Nucl Med Commun* **2017**, *38*, 91–98, doi:[10.1097/MNM.0000000000000606](https://doi.org/10.1097/MNM.0000000000000606). | Original study not in the field of interest |
| Giesel, F.L.; Hadaschik, B.; Cardinale, J.; Radtke, J.; Vinsensia, M.; Lehnert, W.; Kesch, C.; Tolstov, Y.; Singer, S.; Grabe, N.; et al. F-18 Labelled PSMA-1007: Biodistribution, Radiation Dosimetry and Histopathological Validation of Tumor Lesions in Prostate Cancer Patients. *Eur J Nucl Med Mol Imaging* **2017**, *44*, 678–688, doi:[10.1007/s00259-016-3573-4](https://doi.org/10.1007/s00259-016-3573-4). | Original study not in the field of interest |
| Li, X.; Rowe, S.P.; Leal, J.P.; Gorin, M.A.; Allaf, M.E.; Ross, A.E.; Pienta, K.J.; Lodge, M.A.; Pomper, M.G. Semiquantitative Parameters in PSMA-Targeted PET Imaging with 18F-DCFPyL: Variability in Normal-Organ Uptake. *J Nucl Med* **2017**, *58*, 942–946, doi:[10.2967/jnumed.116.179739](https://doi.org/10.2967/jnumed.116.179739). | Original study not in the field of interest |
| Green, M.A.; Eitel, J.A.; Fletcher, J.W.; Mathias, C.J.; Tann, M.A.; Gardner, T.; Koch, M.O.; Territo, W.; Polson, H.; Hutchins, G.D. Estimation of Radiation Dosimetry for 68Ga-HBED-CC (PSMA-11) in Patients with Suspected Recurrence of Prostate Cancer. *Nucl Med Biol* **2017**, *46*, 32–35, doi:[10.1016/j.nucmedbio.2016.11.002](https://doi.org/10.1016/j.nucmedbio.2016.11.002). | Original study not in the field of interest |
| Zacho, H.D.; Nielsen, J.B.; Dettmann, K.; Haberkorn, U.; Petersen, L.J. Incidental Detection of Thyroid Metastases From Renal Cell Carcinoma Using 68Ga-PSMA PET/CT to Assess Prostate Cancer Recurrence. *Clin Nucl Med* **2017**, *42*, 221–222, doi:[10.1097/RLU.0000000000001522](https://doi.org/10.1097/RLU.0000000000001522). | Case report in the field of interest |
| Umbricht, C.A.; Benešová, M.; Schmid, R.M.; Türler, A.; Schibli, R.; van der Meulen, N.P.; Müller, C. 44Sc-PSMA-617 for Radiotheragnostics in Tandem with 177Lu-PSMA-617-Preclinical Investigations in Comparison with 68Ga-PSMA-11 and 68Ga-PSMA-617. *EJNMMI Res* **2017**, *7*, 9, doi:[10.1186/s13550-017-0257-4](https://doi.org/10.1186/s13550-017-0257-4). | Original study not in the field of interest |
| Siva, S.; Callahan, J.; Pryor, D.; Martin, J.; Lawrentschuk, N.; Hofman, M.S. Utility of 68 Ga Prostate Specific Membrane Antigen - Positron Emission Tomography in Diagnosis and Response Assessment of Recurrent Renal Cell Carcinoma. *J Med Imaging Radiat Oncol* **2017**, *61*, 372–378, doi:[10.1111/1754-9485.12590](https://doi.org/10.1111/1754-9485.12590). | Review in the field of interest |
| Noto, B.; Büther, F.; Auf der Springe, K.; Avramovic, N.; Heindel, W.; Schäfers, M.; Allkemper, T.; Stegger, L. Impact of PET Acquisition Durations on Image Quality and Lesion Detectability in Whole-Body 68Ga-PSMA PET-MRI. *EJNMMI Res* **2017**, *7*, 12, doi:[10.1186/s13550-017-0261-8](https://doi.org/10.1186/s13550-017-0261-8). | Original study not in the field of interest |
| Ferro-Flores, G.; Luna-Gutiérrez, M.; Ocampo-García, B.; Santos-Cuevas, C.; Azorín-Vega, E.; Jiménez-Mancilla, N.; Orocio-Rodríguez, E.; Davanzo, J.; García-Pérez, F.O. Clinical Translation of a PSMA Inhibitor for 99mTc-Based SPECT. *Nucl Med Biol* **2017**, *48*, 36–44, doi:[10.1016/j.nucmedbio.2017.01.012](https://doi.org/10.1016/j.nucmedbio.2017.01.012). | Original study not in the field of interest |
| Young, J.D.; Abbate, V.; Imberti, C.; Meszaros, L.K.; Ma, M.T.; Terry, S.Y.A.; Hider, R.C.; Mullen, G.E.; Blower, P.J. 68Ga-THP-PSMA: A PET Imaging Agent for Prostate Cancer Offering Rapid, Room-Temperature, 1-Step Kit-Based Radiolabeling. *J Nucl Med* **2017**, *58*, 1270–1277, doi:[10.2967/jnumed.117.191882](https://doi.org/10.2967/jnumed.117.191882). | Original study not in the field of interest |
| Kelly, J.M.; Amor-Coarasa, A.; Nikolopoulou, A.; Wüstemann, T.; Barelli, P.; Kim, D.; Williams, C.; Zheng, X.; Bi, C.; Hu, B.; et al. Dual-Target Binding Ligands with Modulated Pharmacokinetics for Endoradiotherapy of Prostate Cancer. *J Nucl Med* **2017**, *58*, 1442–1449, doi:[10.2967/jnumed.116.188722](https://doi.org/10.2967/jnumed.116.188722). | Original study not in the field of interest |
| Kirchner, J.; Schaarschmidt, B.M.; Sawicki, L.M.; Heusch, P.; Hautzel, H.; Ermert, J.; Rabenalt, R.; Antoch, G.; Buchbender, C. Evaluation of Practical Interpretation Hurdles in 68Ga-PSMA PET/CT in 55 Patients: Physiological Tracer Distribution and Incidental Tracer Uptake. *Clin Nucl Med* **2017**, *42*, e322–e327, doi:[10.1097/RLU.0000000000001672](https://doi.org/10.1097/RLU.0000000000001672). | Original study not in the field of interest |
| Jilg, C.A.; Drendel, V.; Rischke, H.C.; Beck, T.; Vach, W.; Schaal, K.; Wetterauer, U.; Schultze-Seemann, W.; Meyer, P.T. Diagnostic Accuracy of Ga-68-HBED-CC-PSMA-Ligand-PET/CT before Salvage Lymph Node Dissection for Recurrent Prostate Cancer. *Theranostics* **2017**, *7*, 1770–1780, doi:[10.7150/thno.18421](https://doi.org/10.7150/thno.18421). | Original study not in the field of interest |
| Cui, C.; Hanyu, M.; Hatori, A.; Zhang, Y.; Xie, L.; Ohya, T.; Fukada, M.; Suzuki, H.; Nagatsu, K.; Jiang, C.; et al. Synthesis and Evaluation of [64Cu]PSMA-617 Targeted for Prostate-Specific Membrane Antigen in Prostate Cancer. *Am J Nucl Med Mol Imaging* **2017**, *7*, 40–52. | Original study not in the field of interest |
| Fendler, W.P.; Stuparu, A.D.; Evans-Axelsson, S.; Lückerath, K.; Wei, L.; Kim, W.; Poddar, S.; Said, J.; Radu, C.G.; Eiber, M.; et al. Establishing 177Lu-PSMA-617 Radioligand Therapy in a Syngeneic Model of Murine Prostate Cancer. *J Nucl Med* **2017**, *58*, 1786–1792, doi:[10.2967/jnumed.117.193359](https://doi.org/10.2967/jnumed.117.193359). | Original study not in the field of interest |
| Rowe, S.P.; Gorin, M.A.; Pomper, M.G. Imaging of Prostate-Specific Membrane Antigen Using [18F]DCFPyL. *PET Clin* **2017**, *12*, 289–296, doi:[10.1016/j.cpet.2017.02.006](https://doi.org/10.1016/j.cpet.2017.02.006). | Review not in the field of interest |
| Fennessy, N.; Lee, J.; Shin, J.; Ho, B.; Ali, S.A.; Paschkewitz, R.; Emmett, L. Frusemide Aids Diagnostic Interpretation of 68 Ga-PSMA Positron Emission Tomography/CT in Men with Prostate Cancer. *J Med Imaging Radiat Oncol* **2017**, *61*, 739–744, doi:[10.1111/1754-9485.12625](https://doi.org/10.1111/1754-9485.12625). | Original study not in the field of interest |
| Bouvet, V.; Wuest, M.; Bailey, J.J.; Bergman, C.; Janzen, N.; Valliant, J.F.; Wuest, F. Targeting Prostate-Specific Membrane Antigen (PSMA) with F-18-Labeled Compounds: The Influence of Prosthetic Groups on Tumor Uptake and Clearance Profile. *Mol Imaging Biol* **2017**, *19*, 923–932, doi:[10.1007/s11307-017-1102-x](https://doi.org/10.1007/s11307-017-1102-x). | Original study not in the field of interest |
| Schmidtke, A.; Läppchen, T.; Weinmann, C.; Bier-Schorr, L.; Keller, M.; Kiefer, Y.; Holland, J.P.; Bartholomä, M.D. Gallium Complexation, Stability, and Bioconjugation of 1,4,7-Triazacyclononane Derived Chelators with Azaheterocyclic Arms. *Inorg Chem* **2017**, *56*, 9097–9110, doi:[10.1021/acs.inorgchem.7b01129](https://doi.org/10.1021/acs.inorgchem.7b01129). | Original study not in the field of interest |
| Baranski, A.-C.; Schäfer, M.; Bauder-Wüst, U.; Wacker, A.; Schmidt, J.; Liolios, C.; Mier, W.; Haberkorn, U.; Eisenhut, M.; Kopka, K.; et al. Improving the Imaging Contrast of 68Ga-PSMA-11 by Targeted Linker Design: Charged Spacer Moieties Enhance the Pharmacokinetic Properties. *Bioconjug Chem* **2017**, *28*, 2485–2492, doi:[10.1021/acs.bioconjchem.7b00458](https://doi.org/10.1021/acs.bioconjchem.7b00458). | Original study not in the field of interest |
| Matteucci, F.; Mezzenga, E.; Caroli, P.; Di Iorio, V.; Sarnelli, A.; Celli, M.; Fantini, L.; Moretti, A.; Galassi, R.; De Giorgi, U.; et al. Reduction of 68Ga-PSMA Renal Uptake with Mannitol Infusion: Preliminary Results. *Eur J Nucl Med Mol Imaging* **2017**, *44*, 2189–2194, doi:[10.1007/s00259-017-3791-4](https://doi.org/10.1007/s00259-017-3791-4). | Original study not in the field of interest |
| Heußer, T.; Mann, P.; Rank, C.M.; Schäfer, M.; Dimitrakopoulou-Strauss, A.; Schlemmer, H.-P.; Hadaschik, B.A.; Kopka, K.; Bachert, P.; Kachelrieß, M.; et al. Investigation of the Halo-Artifact in 68Ga-PSMA-11-PET/MRI. *PLoS One* **2017**, *12*, e0183329, doi:[10.1371/journal.pone.0183329](https://doi.org/10.1371/journal.pone.0183329). | Original study not in the field of interest |
| Werner, R.A.; Sheikhbahaei, S.; Jones, K.M.; Javadi, M.S.; Solnes, L.B.; Ross, A.E.; Allaf, M.E.; Pienta, K.J.; Lapa, C.; Buck, A.K.; et al. Patterns of Uptake of Prostate-Specific Membrane Antigen (PSMA)-Targeted 18F-DCFPyL in Peripheral Ganglia. *Ann Nucl Med* **2017**, *31*, 696–702, doi:[10.1007/s12149-017-1201-4](https://doi.org/10.1007/s12149-017-1201-4). | Original study not in the field of interest |
| Ried, K.; Eng, P.; Sali, A. Screening for Circulating Tumour Cells Allows Early Detection of Cancer and Monitoring of Treatment Effectiveness: An Observational Study. *Asian Pac J Cancer Prev* **2017**, *18*, 2275–2285, doi:[10.22034/APJCP.2017.18.8.2275](https://doi.org/10.22034/APJCP.2017.18.8.2275). | Original study not in the field of interest |
| Nadebaum, D.P.; Hofman, M.S.; Mitchell, C.A.; Siva, S.; Hicks, R.J. Oligometastatic Renal Cell Carcinoma With Sarcomatoid Differentiation Demonstrating Variable Imaging Phenotypes on 68Ga-PSMA and 18F-FDG PET/CT: A Case Report and Review of the Literature. *Clin Genitourin Cancer* **2017**, S1558-7673(17)30250-1, doi:[10.1016/j.clgc.2017.08.009](https://doi.org/10.1016/j.clgc.2017.08.009). | Case report and review of literature in the field of interest |
| Kratochwil, C.; Schmidt, K.; Afshar-Oromieh, A.; Bruchertseifer, F.; Rathke, H.; Morgenstern, A.; Haberkorn, U.; Giesel, F.L. Targeted Alpha Therapy of MCRPC: Dosimetry Estimate of 213Bismuth-PSMA-617. *Eur J Nucl Med Mol Imaging* **2018**, *45*, 31–37, doi:[10.1007/s00259-017-3817-y](https://doi.org/10.1007/s00259-017-3817-y). | Original study not in the field of interest |
| Gaertner, F.C.; Halabi, K.; Ahmadzadehfar, H.; Kürpig, S.; Eppard, E.; Kotsikopoulos, C.; Liakos, N.; Bundschuh, R.A.; Strunk, H.; Essler, M. Uptake of PSMA-Ligands in Normal Tissues Is Dependent on Tumor Load in Patients with Prostate Cancer. *Oncotarget* **2017**, *8*, 55094–55103, doi:[10.18632/oncotarget.19049](https://doi.org/10.18632/oncotarget.19049). | Original study not in the field of interest |
| Dam, J.H.; Olsen, B.B.; Baun, C.; Høilund-Carlsen, P.F.; Thisgaard, H. A PSMA Ligand Labeled with Cobalt-55 for PET Imaging of Prostate Cancer. *Mol Imaging Biol* **2017**, *19*, 915–922, doi:[10.1007/s11307-017-1121-7](https://doi.org/10.1007/s11307-017-1121-7). | Original study not in the field of interest |
| Saadat, S.; Tie, B.; Wood, S.; Vela, I.; Rhee, H. Imaging Tumour Thrombus of Clear Cell Renal Cell Carcinoma: FDG PET or PSMA PET? Direct in Vivo Comparison of Two Technologies. *Urol Case Rep* **2018**, *16*, 4–5, doi:[10.1016/j.eucr.2017.09.010](https://doi.org/10.1016/j.eucr.2017.09.010). | Case report in the field of interest |
| Freitag, M.T.; Kesch, C.; Cardinale, J.; Flechsig, P.; Floca, R.; Eiber, M.; Bonekamp, D.; Radtke, J.P.; Kratochwil, C.; Kopka, K.; et al. Simultaneous Whole-Body 18F-PSMA-1007-PET/MRI with Integrated High-Resolution Multiparametric Imaging of the Prostatic Fossa for Comprehensive Oncological Staging of Patients with Prostate Cancer: A Pilot Study. *Eur J Nucl Med Mol Imaging* **2018**, *45*, 340–347, doi:[10.1007/s00259-017-3854-6](https://doi.org/10.1007/s00259-017-3854-6). | Original study not in the field of interest |
| Campbell, S.P.; Baras, A.S.; Ball, M.W.; Kates, M.; Hahn, N.M.; Bivalacqua, T.J.; Johnson, M.H.; Pomper, M.G.; Allaf, M.E.; Rowe, S.P.; et al. Low Levels of PSMA Expression Limit the Utility of 18F-DCFPyL PET/CT for Imaging Urothelial Carcinoma. *Ann Nucl Med* **2018**, *32*, 69–74, doi:[10.1007/s12149-017-1216-x](https://doi.org/10.1007/s12149-017-1216-x). | Original study not in the field of interest |
| Nawaz, S.; Mullen, G.E.D.; Sunassee, K.; Bordoloi, J.; Blower, P.J.; Ballinger, J.R. Simple, Mild, One-Step Labelling of Proteins with Gallium-68 Using a Tris(Hydroxypyridinone) Bifunctional Chelator: A 68Ga-THP-ScFv Targeting the Prostate-Specific Membrane Antigen. *EJNMMI Res* **2017**, *7*, 86, doi:[10.1186/s13550-017-0336-6](https://doi.org/10.1186/s13550-017-0336-6). | Original study not in the field of interest |
| Gourni, E.; Del Pozzo, L.; Bartholomä, M.; Kiefer, Y.; T Meyer, P.; Maecke, H.R.; Holland, J.P. Radiochemistry and Preclinical PET Imaging of 68Ga-Desferrioxamine Radiotracers Targeting Prostate-Specific Membrane Antigen. *Mol Imaging* **2017**, *16*, 1536012117737010, doi:[10.1177/1536012117737010](https://doi.org/10.1177/1536012117737010). | Original study not in the field of interest |
| Eppard, E.; de la Fuente, A.; Benešová, M.; Khawar, A.; Bundschuh, R.A.; Gärtner, F.C.; Kreppel, B.; Kopka, K.; Essler, M.; Rösch, F. Clinical Translation and First In-Human Use of [44Sc]Sc-PSMA-617 for PET Imaging of Metastasized Castrate-Resistant Prostate Cancer. *Theranostics* **2017**, *7*, 4359–4369, doi:[10.7150/thno.20586](https://doi.org/10.7150/thno.20586). | Original study not in the field of interest |
| Udovicich, C.; Perera, M.; Hofman, M.S.; Siva, S.; Del Rio, A.; Murphy, D.G.; Lawrentschuk, N. 68Ga-Prostate-Specific Membrane Antigen-Positron Emission Tomography/Computed Tomography in Advanced Prostate Cancer: Current State and Future Trends. *Prostate Int* **2017**, *5*, 125–129, doi:[10.1016/j.prnil.2017.02.003](https://doi.org/10.1016/j.prnil.2017.02.003). | Review not in the field of interest |
| Jochumsen, M.R.; Gormsen, L.C.; Nielsen, G.L. 68Ga-PSMA Avid Primary Adenocarcinoma of the Lung With Complementary Low 18F-FDG Uptake. *Clin Nucl Med* **2018**, *43*, 117–119, doi:[10.1097/RLU.0000000000001935](https://doi.org/10.1097/RLU.0000000000001935). | Case report not in the field of interest |
| Will, L.; Giesel, F.L.; Freitag, M.T.; Berger, A.K.; Mier, W.; Kopka, K.; Koerber, S.A.; Rathke, H.; Kremer, C.; Kratochwil, C.; et al. Integration of CT Urography Improves Diagnostic Confidence of 68Ga-PSMA-11 PET/CT in Prostate Cancer Patients. *Cancer Imaging* **2017**, *17*, 30, doi:[10.1186/s40644-017-0132-6](https://doi.org/10.1186/s40644-017-0132-6). | Review not in the field of interest |
| Giesel, F.L.; Will, L.; Lawal, I.; Lengana, T.; Kratochwil, C.; Vorster, M.; Neels, O.; Reyneke, F.; Haberkon, U.; Kopka, K.; et al. Intraindividual Comparison of 18F-PSMA-1007 and 18F-DCFPyL PET/CT in the Prospective Evaluation of Patients with Newly Diagnosed Prostate Carcinoma: A Pilot Study. *J Nucl Med* **2018**, *59*, 1076–1080, doi:[10.2967/jnumed.117.204669](https://doi.org/10.2967/jnumed.117.204669). | Original study not in the field of interest |
| Lawhn-Heath, C.; Flavell, R.R.; Korenchan, D.E.; Deller, T.; Lake, S.; Carroll, P.R.; Hope, T.A. Scatter Artifact with Ga-68-PSMA-11 PET: Severity Reduced With Furosemide Diuresis and Improved Scatter Correction. *Mol Imaging* **2018**, *17*, 1536012118811741, doi:[10.1177/1536012118811741](https://doi.org/10.1177/1536012118811741). | Original study not in the field of interest |
| Damle, N.A.; Bal, C.; Singh, T.P.; Gupta, R.; Reddy, S.; Kumar, R.; Tripathi, M. Anaplastic Thyroid Carcinoma on 68 Ga-PSMA PET/CT: Opening New Frontiers. *Eur J Nucl Med Mol Imaging* **2018**, *45*, 667–668, doi:[10.1007/s00259-017-3904-0](https://doi.org/10.1007/s00259-017-3904-0). | Case report not in the field of interest |
| ùDerlin, T.; Schmuck, S.; Juhl, C.; Teichert, S.; Zörgiebel, J.; Wester, H.-J.; Schneefeld, S.M.; Walte, A.C.A.; Thackeray, J.T.; Ross, T.L.; et al. Imaging Characteristics and First Experience of [68Ga]THP-PSMA, a Novel Probe for Rapid Kit-Based Ga-68 Labeling and PET Imaging: Comparative Analysis with [68Ga]PSMA I&T. *Mol Imaging Biol* **2018**, *20*, 650–658, doi:[10.1007/s11307-018-1160-8](https://doi.org/10.1007/s11307-018-1160-8). | Original study not in the field of interest |
| Zha, Z.; Ploessl, K.; Choi, S.R.; Wu, Z.; Zhu, L.; Kung, H.F. Synthesis and Evaluation of a Novel Urea-Based 68Ga-Complex for Imaging PSMA Binding in Tumor. *Nucl Med Biol* **2018**, *59*, 36–47, doi:[10.1016/j.nucmedbio.2017.12.007](https://doi.org/10.1016/j.nucmedbio.2017.12.007). | Original study not in the field of interest |
| Plyku, D.; Mena, E.; Rowe, S.P.; Lodge, M.A.; Szabo, Z.; Cho, S.Y.; Pomper, M.G.; Sgouros, G.; Hobbs, R.F. Combined Model-Based and Patient-Specific Dosimetry for 18F-DCFPyL, a PSMA-Targeted PET Agent. *Eur J Nucl Med Mol Imaging* **2018**, *45*, 989–998, doi:[10.1007/s00259-018-3939-x](https://doi.org/10.1007/s00259-018-3939-x). | Original study not in the field of interest |
| Khawar, A.; Eppard, E.; Sinnes, J.P.; Roesch, F.; Ahmadzadehfar, H.; Kürpig, S.; Meisenheimer, M.; Gaertner, F.C.; Essler, M.; Bundschuh, R.A. [44Sc]Sc-PSMA-617 Biodistribution and Dosimetry in Patients With Metastatic Castration-Resistant Prostate Carcinoma. *Clin Nucl Med* **2018**, *43*, 323–330, doi:[10.1097/RLU.0000000000002003](https://doi.org/10.1097/RLU.0000000000002003). | Original study not in the field of interest |
| Filss, C.; Heinzel, A.; Miiller, B.; Vogg, A.T.J.; Langen, K.-J.; Mottaghy, F.M. Relevant Tumor Sink Effect in Prostate Cancer Patients Receiving 177Lu-PSMA-617 Radioligand Therapy. *Nuklearmedizin* **2018**, *57*, 19–25, doi:[10.3413/Nukmed-0937-17-10](https://doi.org/10.3413/Nukmed-0937-17-10). | Original study not in the field of interest |
| Rahbar, K.; Afshar-Oromieh, A.; Bögemann, M.; Wagner, S.; Schäfers, M.; Stegger, L.; Weckesser, M. 18F-PSMA-1007 PET/CT at 60 and 120 Minutes in Patients with Prostate Cancer: Biodistribution, Tumour Detection and Activity Kinetics. *Eur J Nucl Med Mol Imaging* **2018**, *45*, 1329–1334, doi:[10.1007/s00259-018-3989-0](https://doi.org/10.1007/s00259-018-3989-0). | Original study not in the field of interest |
| Salas Fragomeni, R.A.; Amir, T.; Sheikhbahaei, S.; Harvey, S.C.; Javadi, M.S.; Solnes, L.B.; Kiess, A.P.; Allaf, M.E.; Pomper, M.G.; Gorin, M.A.; et al. Imaging of Nonprostate Cancers Using PSMA-Targeted Radiotracers: Rationale, Current State of the Field, and a Call to Arms. *J Nucl Med* **2018**, *59*, 871–877, doi:[10.2967/jnumed.117.203570](https://doi.org/10.2967/jnumed.117.203570). | Review not in the field of interest |
| Läppchen, T.; Kiefer, Y.; Holland, J.P.; Bartholomä, M.D. In Vitro and in Vivo Evaluation of the Bifunctional Chelator NODIA-Me in Combination with a Prostate-Specific Membrane Antigen Targeting Vector. *Nucl Med Biol* **2018**, *60*, 45–54, doi:[10.1016/j.nucmedbio.2018.03.002](https://doi.org/10.1016/j.nucmedbio.2018.03.002). | Original article not in the field of interest |
| Robu, S.; Schmidt, A.; Eiber, M.; Schottelius, M.; Günther, T.; Hooshyar Yousefi, B.; Schwaiger, M.; Wester, H.-J. Synthesis and Preclinical Evaluation of Novel 18F-Labeled Glu-Urea-Glu-Based PSMA Inhibitors for Prostate Cancer Imaging: A Comparison with 18F-DCFPyl and 18F-PSMA-1007. *EJNMMI Res* **2018**, *8*, 30, doi:[10.1186/s13550-018-0382-8](https://doi.org/10.1186/s13550-018-0382-8). | Original article not in the field of interest |
| Moon, S.-H.; Hong, M.K.; Kim, Y.J.; Lee, Y.-S.; Lee, D.S.; Chung, J.-K.; Jeong, J.M. Development of a Ga-68 Labeled PET Tracer with Short Linker for Prostate-Specific Membrane Antigen (PSMA) Targeting. *Bioorg Med Chem* **2018**, *26*, 2501–2507, doi:[10.1016/j.bmc.2018.04.014](https://doi.org/10.1016/j.bmc.2018.04.014). | Original article not in the field of interest |
| Khawar, A.; Eppard, E.; Sinnes, J.P.; Roesch, F.; Ahmadzadehfar, H.; Kürpig, S.; Meisenheimer, M.; Gaertner, F.C.; Essler, M.; Bundschuh, R.A. Prediction of Normal Organ Absorbed Doses for [177Lu]Lu-PSMA-617 Using [44Sc]Sc-PSMA-617 Pharmacokinetics in Patients With Metastatic Castration Resistant Prostate Carcinoma. *Clin Nucl Med* **2018**, *43*, 486–491, doi:[10.1097/RLU.0000000000002102](https://doi.org/10.1097/RLU.0000000000002102). | Original article not in the field of interest |
| Sarikaya, I.; Elgazzar, A.H.; Alfeeli, M.A.; Sarikaya, A. Can Gallium-68 Prostate-Specific Membrane Antigen Ligand Be a Potential Radiotracer for Renal Cortical Positron Emission Tomography Imaging? *World J Nucl Med* **2018**, *17*, 126–129, doi:[10.4103/wjnm.WJNM_35_17](https://doi.org/10.4103/wjnm.WJNM_35_17). | Case report not in the field of interest |
| Sevcenco, S.; Klingler, H.C.; Eredics, K.; Friedl, A.; Schneeweiss, J.; Knoll, P.; Kunit, T.; Lusuardi, L.; Mirzaei, S. Application of Cu-64 NODAGA-PSMA PET in Prostate Cancer. *Adv Ther* **2018**, *35*, 779–784, doi:[10.1007/s12325-018-0711-3](https://doi.org/10.1007/s12325-018-0711-3). | Original article not in the field of interest |
| Wang, Y.; Shao, G.; Wu, J.; Cui, C.; Zang, S.; Qiu, F.; Jia, R.; Wang, Z.; Wang, F. Preparation of 68Ga-PSMA-11 with a Synthesis Module for Micro PET-CT Imaging of PSMA Expression during Prostate Cancer Progression. *Contrast Media Mol Imaging* **2018**, *2018*, 8046541, doi:[10.1155/2018/8046541](https://doi.org/10.1155/2018/8046541). | Original article not in the field of interest |
| Wangerin, K.A.; Baratto, L.; Khalighi, M.M.; Hope, T.A.; Gulaka, P.K.; Deller, T.W.; Iagaru, A.H. Clinical Evaluation of 68Ga-PSMA-II and 68Ga-RM2 PET Images Reconstructed With an Improved Scatter Correction Algorithm. *AJR Am J Roentgenol* **2018**, *211*, 655–660, doi:[10.2214/AJR.17.19356](https://doi.org/10.2214/AJR.17.19356). | Original article not in the field of interest |
| Kuo, H.-T.; Pan, J.; Zhang, Z.; Lau, J.; Merkens, H.; Zhang, C.; Colpo, N.; Lin, K.-S.; Bénard, F. Effects of Linker Modification on Tumor-to-Kidney Contrast of 68Ga-Labeled PSMA-Targeted Imaging Probes. *Mol Pharm* **2018**, *15*, 3502–3511, doi:[10.1021/acs.molpharmaceut.8b00499](https://doi.org/10.1021/acs.molpharmaceut.8b00499). | Original article not in the field of interest |
| Cantiello, F.; Crocerossa, F.; Russo, G.I.; Gangemi, V.; Ferro, M.; Vartolomei, M.D.; Lucarelli, G.; Mirabelli, M.; Scafuro, C.; Ucciero, G.; et al. Comparison Between 64Cu-PSMA-617 PET/CT and 18F-Choline PET/CT Imaging in Early Diagnosis of Prostate Cancer Biochemical Recurrence. *Clin Genitourin Cancer* **2018**, *16*, 385–391, doi:[10.1016/j.clgc.2018.05.014](https://doi.org/10.1016/j.clgc.2018.05.014). | Original article not in the field of interest |
| Perveen, G.; Arora, G.; Damle, N.A.; Prabhu, M.; Arora, S.; Tripathi, M.; Bal, C.; Kumar, P.; Kumar, R.; Singh, P.; et al. Can Early Dynamic Positron Emission Tomography/Computed Tomography Obviate the Need for Postdiuresis Image in 68Ga-PSMA-HBED-CC Scan for Evaluation of Prostate Adenocarcinoma? *Indian J Nucl Med* **2018**, *33*, 202–208, doi:[10.4103/ijnm.IJNM_32_18](https://doi.org/10.4103/ijnm.IJNM_32_18). | Original article not in the field of interest |
| Prado Júnior, L.M.; Marino, F.M.; Barra, R.; do Prado, L.F.M.; Barra Sobrinho, A. One-Year Experience with 68Ga-PSMA PET/CT: Applications and Results in Biochemical Recurrence of Prostate Cancer. *Radiol Bras* **2018**, *51*, 151–155, doi:[10.1590/0100-3984.2017.0008](https://doi.org/10.1590/0100-3984.2017.0008). | Original article not in the field of interest |
| Wurzer, A.; Pollmann, J.; Schmidt, A.; Reich, D.; Wester, H.-J.; Notni, J. Molar Activity of Ga-68 Labeled PSMA Inhibitor Conjugates Determines PET Imaging Results. *Mol Pharm* **2018**, *15*, 4296–4302, doi:[10.1021/acs.molpharmaceut.8b00602](https://doi.org/10.1021/acs.molpharmaceut.8b00602). | Original article not in the field of interest |
| Sahoo, M.K.; Ahlawat, K.; Yadav, R.; Gajendra, S. Schmorl’s Node: Confusion Still Persists in 68Ga-Prostate-Specific Membrane Antigen Ligand PET/CT. *Clin Nucl Med* **2018**, *43*, 679–681, doi:[10.1097/RLU.0000000000002196](https://doi.org/10.1097/RLU.0000000000002196). | Case report not in the field of interest |
| Giesel, F.L.; Kratochwil, C.; Lindner, T.; Marschalek, M.M.; Loktev, A.; Lehnert, W.; Debus, J.; Jäger, D.; Flechsig, P.; Altmann, A.; et al. 68Ga-FAPI PET/CT: Biodistribution and Preliminary Dosimetry Estimate of 2 DOTA-Containing FAP-Targeting Agents in Patients with Various Cancers. *J Nucl Med* **2019**, *60*, 386–392, doi:[10.2967/jnumed.118.215913](https://doi.org/10.2967/jnumed.118.215913). | Original article not in the field of interest |
| Schmidt, A.; Wirtz, M.; Färber, S.F.; Osl, T.; Beck, R.; Schottelius, M.; Schwaiger, M.; Wester, H.-J. Effect of Carbohydration on the Theranostic Tracer PSMA I&T. *ACS Omega* **2018**, *3*, 8278–8287, doi:[10.1021/acsomega.8b00790](https://doi.org/10.1021/acsomega.8b00790). | Original article not in the field of interest |
| Zang, J.; Fan, X.; Wang, H.; Liu, Q.; Wang, J.; Li, H.; Li, F.; Jacobson, O.; Niu, G.; Zhu, Z.; et al. First-in-Human Study of 177Lu-EB-PSMA-617 in Patients with Metastatic Castration-Resistant Prostate Cancer. *Eur J Nucl Med Mol Imaging* **2019**, *46*, 148–158, doi:[10.1007/s00259-018-4096-y](https://doi.org/10.1007/s00259-018-4096-y). | Original article not in the field of interest |
| Rousseau, E.; Lau, J.; Kuo, H.-T.; Zhang, Z.; Merkens, H.; Hundal-Jabal, N.; Colpo, N.; Lin, K.-S.; Bénard, F. Monosodium Glutamate Reduces 68Ga-PSMA-11 Uptake in Salivary Glands and Kidneys in a Preclinical Prostate Cancer Model. *J Nucl Med* **2018**, *59*, 1865–1868, doi:[10.2967/jnumed.118.215350](https://doi.org/10.2967/jnumed.118.215350). | Original article not in the field of interest |
| Evangelista, L.; Basso, U.; Maruzzo, M.; Novara, G. The Role of Radiolabeled Prostate-Specific Membrane Antigen Positron Emission Tomography/Computed Tomography for the Evaluation of Renal Cancer. *Eur Urol Focus* **2020**, *6*, 146–150, doi:[10.1016/j.euf.2018.08.004](https://doi.org/10.1016/j.euf.2018.08.004). | Review in the field of interest |
| Wirtz, M.; Schmidt, A.; Schottelius, M.; Robu, S.; Günther, T.; Schwaiger, M.; Wester, H.-J. Synthesis and in Vitro and in Vivo Evaluation of Urea-Based PSMA Inhibitors with Increased Lipophilicity. *EJNMMI Res* **2018**, *8*, 84, doi:[10.1186/s13550-018-0440-2](https://doi.org/10.1186/s13550-018-0440-2). | Original article not in the field of interest |
| Demirci, E.; Toklu, T.; Yeyin, N.; Ocak, M.; Alan-Selcuk, N.; Araman, A.; Kabasakal, L. ESTIMATION OF THE ORGAN ABSORBED DOSES AND EFFECTIVE DOSE FROM 68Ga-PSMA-11 PET SCAN. *Radiat Prot Dosimetry* **2018**, *182*, 518–524, doi:[10.1093/rpd/ncy111](https://doi.org/10.1093/rpd/ncy111). | Original article not in the field of interest |
| Sathekge, M.; Bruchertseifer, F.; Knoesen, O.; Reyneke, F.; Lawal, I.; Lengana, T.; Davis, C.; Mahapane, J.; Corbett, C.; Vorster, M.; et al. 225Ac-PSMA-617 in Chemotherapy-Naive Patients with Advanced Prostate Cancer: A Pilot Study. *Eur J Nucl Med Mol Imaging* **2019**, *46*, 129–138, doi:[10.1007/s00259-018-4167-0](https://doi.org/10.1007/s00259-018-4167-0). | Original article not in the field of interest |
| Schottelius, M.; Wurzer, A.; Wissmiller, K.; Beck, R.; Koch, M.; Gorpas, D.; Notni, J.; Buckle, T.; van Oosterom, M.N.; Steiger, K.; et al. Synthesis and Preclinical Characterization of the PSMA-Targeted Hybrid Tracer PSMA-I&F for Nuclear and Fluorescence Imaging of Prostate Cancer. *J Nucl Med* **2019**, *60*, 71–78, doi:[10.2967/jnumed.118.212720](https://doi.org/10.2967/jnumed.118.212720). | Original article not in the field of interest |
| Violet, J.; Jackson, P.; Ferdinandus, J.; Sandhu, S.; Akhurst, T.; Iravani, A.; Kong, G.; Kumar, A.R.; Thang, S.P.; Eu, P.; et al. Dosimetry of 177Lu-PSMA-617 in Metastatic Castration-Resistant Prostate Cancer: Correlations Between Pretherapeutic Imaging and Whole-Body Tumor Dosimetry with Treatment Outcomes. *J Nucl Med* **2019**, *60*, 517–523, doi:[10.2967/jnumed.118.219352](https://doi.org/10.2967/jnumed.118.219352). | Original article not in the field of interest |
| Li, J.; Xu, R.; Kim, C.K.; Bénard, F.; Kapoor, A.; Bauman, G.; Zukotynski, K.A. 18F-DCFPyL PET/CT in Oncocytoma. *Clin Nucl Med* **2018**, *43*, 921–924, doi:[10.1097/RLU.0000000000002301](https://doi.org/10.1097/RLU.0000000000002301). | Case report not in the field of interest |
| Amor-Coarasa, A.; Kelly, J.M.; Ponnala, S.; Nikolopoulou, A.; Williams, C.; Babich, J.W. 66Ga: A Novelty or a Valuable Preclinical Screening Tool for the Design of Targeted Radiopharmaceuticals? *Molecules* **2018**, *23*, E2575, doi:[10.3390/molecules23102575](https://doi.org/10.3390/molecules23102575). | Original article not in the field of interest |
| Umbricht, C.A.; Benešová, M.; Hasler, R.; Schibli, R.; van der Meulen, N.P.; Müller, C. Design and Preclinical Evaluation of an Albumin-Binding PSMA Ligand for 64Cu-Based PET Imaging. *Mol Pharm* **2018**, *15*, 5556–5564, doi:[10.1021/acs.molpharmaceut.8b00712](https://doi.org/10.1021/acs.molpharmaceut.8b00712). | Original article not in the field of interest |
| Malik, D.; Sood, A.; Mittal, B.R.; Singh, H.; Basher, R.K.; Shukla, J.; Bhattacharya, A.; Singh, S.K. Nonspecific Uptake of 68Ga-Prostate-Specific Membrane Antigen in Diseases Other than Prostate Malignancy on Positron Emission Tomography/Computed Tomography Imaging: A Pictorial Assay and Review of Literature. *Indian J Nucl Med* **2018**, *33*, 317–325, doi:[10.4103/ijnm.IJNM_81_18](https://doi.org/10.4103/ijnm.IJNM_81_18). | Original article not in the field of interest |
| Joraku, A.; Hatano, K.; Kawai, K.; Kandori, S.; Kojima, T.; Fukumitsu, N.; Isobe, T.; Mori, Y.; Sakata, M.; Hara, T.; et al. Phase I/IIa PET Imaging Study with 89zirconium Labeled Anti-PSMA Minibody for Urological Malignancies. *Ann Nucl Med* **2019**, *33*, 119–127, doi:[10.1007/s12149-018-1312-6](https://doi.org/10.1007/s12149-018-1312-6). | Original article not in the field of interest |
| Behr, S.C.; Aggarwal, R.; VanBrocklin, H.F.; Flavell, R.R.; Gao, K.; Small, E.J.; Blecha, J.; Jivan, S.; Hope, T.A.; Simko, J.P.; et al. Phase I Study of CTT1057, an 18F-Labeled Imaging Agent with Phosphoramidate Core Targeting Prostate-Specific Membrane Antigen in Prostate Cancer. *J Nucl Med* **2019**, *60*, 910–916, doi:[10.2967/jnumed.118.220715](https://doi.org/10.2967/jnumed.118.220715). | Original article not in the field of interest |
| Yordanova, A.; Linden, P.; Hauser, S.; Meisenheimer, M.; Kürpig, S.; Feldmann, G.; Gaertner, F.C.; Essler, M.; Ahmadzadehfar, H. Outcome and Safety of Rechallenge [177Lu]Lu-PSMA-617 in Patients with Metastatic Prostate Cancer. *Eur J Nucl Med Mol Imaging* **2019**, *46*, 1073–1080, doi:[10.1007/s00259-018-4222-x](https://doi.org/10.1007/s00259-018-4222-x). | Original article not in the field of interest |
| Santos-Cuevas, C.; Ferro-Flores, G.; García-Pérez, F.O.; Jiménez-Mancilla, N.; Ramírez-Nava, G.; Ocampo-García, B.; Luna-Gutiérrez, M.; Azorín-Vega, E.; Davanzo, J.; Soldevilla-Gallardo, I. 177Lu-DOTA-HYNIC-Lys(Nal)-Urea-Glu: Biokinetics, Dosimetry, and Evaluation in Patients with Advanced Prostate Cancer. *Contrast Media Mol Imaging* **2018**, *2018*, 5247153, doi:[10.1155/2018/5247153](https://doi.org/10.1155/2018/5247153). | Original article not in the field of interest |
| Kelly, J.M.; Amor-Coarasa, A.; Ponnala, S.; Nikolopoulou, A.; Williams, C.; DiMagno, S.G.; Babich, J.W. Albumin-Binding PSMA Ligands: Implications for Expanding the Therapeutic Window. *J Nucl Med* **2019**, *60*, 656–663, doi:[10.2967/jnumed.118.221150](https://doi.org/10.2967/jnumed.118.221150). | Original article not in the field of interest |
| Shetty, D.; Patel, D.; Le, K.; Bui, C.; Mansberg, R. Pitfalls in Gallium-68 PSMA PET/CT Interpretation-A Pictorial Review. *Tomography* **2018**, *4*, 182–193, doi:[10.18383/j.tom.2018.00021](https://doi.org/10.18383/j.tom.2018.00021). | Review not in the field of interest |
| Dowling, M.; Samuelson, J.; Fadl-Alla, B.; Pondenis, H.C.; Byrum, M.; Barger, A.M.; Fan, T.M. Overexpression of Prostate Specific Membrane Antigen by Canine Hemangiosarcoma Cells Provides Opportunity for the Molecular Detection of Disease Burdens within Hemorrhagic Body Cavity Effusions. *PLoS One* **2019**, *14*, e0210297, doi:[10.1371/journal.pone.0210297](https://doi.org/10.1371/journal.pone.0210297). | Original article not in the field of interest |
| Ahn, T.; Roberts, M.J.; Abduljabar, A.; Joshi, A.; Perera, M.; Rhee, H.; Wood, S.; Vela, I. A Review of Prostate-Specific Membrane Antigen (PSMA) Positron Emission Tomography (PET) in Renal Cell Carcinoma (RCC). *Mol Imaging Biol* **2019**, *21*, 799–807, doi:[10.1007/s11307-018-01307-0](https://doi.org/10.1007/s11307-018-01307-0). | Review in the field of interest |
| Sandgren, K.; Johansson, L.; Axelsson, J.; Jonsson, J.; Ögren, M.; Ögren, M.; Andersson, M.; Strandberg, S.; Nyholm, T.; Riklund, K.; et al. Radiation Dosimetry of [68Ga]PSMA-11 in Low-Risk Prostate Cancer Patients. *EJNMMI Phys* **2019**, *6*, 2, doi:[10.1186/s40658-018-0239-2](https://doi.org/10.1186/s40658-018-0239-2). | Original article not in the field of interest |
| Lütje, S.; Franssen, G.M.; Herrmann, K.; Boerman, O.C.; Rijpkema, M.; Gotthardt, M.; Heskamp, S. In Vitro and In Vivo Characterization of an 18F-AlF-Labeled PSMA Ligand for Imaging of PSMA-Expressing Xenografts. *J Nucl Med* **2019**, *60*, 1017–1022, doi:[10.2967/jnumed.118.218941](https://doi.org/10.2967/jnumed.118.218941). | Original article not in the field of interest |
| Rowe, S.P.; Gorin, M.A.; Pomper, M.G. Imaging of Prostate-Specific Membrane Antigen with Small-Molecule PET Radiotracers: From the Bench to Advanced Clinical Applications. *Annu Rev Med* **2019**, *70*, 461–477, doi:[10.1146/annurev-med-062117-073027](https://doi.org/10.1146/annurev-med-062117-073027). | Review not in the field of interest |
| Lindenberg, L.; Mena, E.; Choyke, P.L.; Bouchelouche, K. PET Imaging in Renal Cancer. *Curr Opin Oncol* **2019**, *31*, 216–221, doi:[10.1097/CCO.0000000000000518](https://doi.org/10.1097/CCO.0000000000000518). | Review in the field of interest |
| Liu, T.; Liu, C.; Xu, X.; Liu, F.; Guo, X.; Li, N.; Wang, X.; Yang, J.; Yang, X.; Zhu, H.; et al. Preclinical Evaluation and Pilot Clinical Study of Al18F-PSMA-BCH for Prostate Cancer PET Imaging. *J Nucl Med* **2019**, *60*, 1284–1292, doi:[10.2967/jnumed.118.221671](https://doi.org/10.2967/jnumed.118.221671). | Original article not in the field of interest |
| McEwan, L.; McBean, R.; Yaxley, J.; Wong, D. Unexpected Significant Findings Non-Related to Prostate Cancer Identified Using Combined Prostate-Specific Membrane Antigen Positron Emission Tomography/CT and Diagnostic CT Scan in Primary Staging for Prostate Cancer. *J Med Imaging Radiat Oncol* **2019**, *63*, 318–323, doi:[10.1111/1754-9485.12864](https://doi.org/10.1111/1754-9485.12864). | Original article not in the field of interest |
| Pianou, N.K.; Stavrou, P.Z.; Vlontzou, E.; Rondogianni, P.; Exarhos, D.N.; Datseris, I.E. More Advantages in Detecting Bone and Soft Tissue Metastases from Prostate Cancer Using 18F-PSMA PET/CT. *Hell J Nucl Med* **2019**, *22*, 6–9, doi:[10.1967/s002449910952](https://doi.org/10.1967/s002449910952). | Editorial not in the field of interest |
| Khawar, A.; Eppard, E.; Roesch, F.; Ahmadzadehfar, H.; Kürpig, S.; Meisenheimer, M.; Gaertner, F.C.; Essler, M.; Bundschuh, R.A. Preliminary Results of Biodistribution and Dosimetric Analysis of [68Ga]Ga-DOTAZOL: A New Zoledronate-Based Bisphosphonate for PET/CT Diagnosis of Bone Diseases. *Ann Nucl Med* **2019**, *33*, 404–413, doi:[10.1007/s12149-019-01348-7](https://doi.org/10.1007/s12149-019-01348-7). | Original article not in the field of interest |
| Seifert, R.; Schafigh, D.; Bögemann, M.; Weckesser, M.; Rahbar, K. Detection of Local Relapse of Prostate Cancer With 18F-PSMA-1007. *Clin Nucl Med* **2019**, *44*, e394–e395, doi:[10.1097/RLU.0000000000002543](https://doi.org/10.1097/RLU.0000000000002543). | Case report not in the field of interest |
| Soeda, F.; Watabe, T.; Naka, S.; Liu, Y.; Horitsugi, G.; Neels, O.C.; Kopka, K.; Tatsumi, M.; Shimosegawa, E.; Giesel, F.L.; et al. Impact of 18F-PSMA-1007 Uptake in Prostate Cancer Using Different Peptide Concentrations: Preclinical PET/CT Study on Mice. *J Nucl Med* **2019**, *60*, 1594–1599, doi:[10.2967/jnumed.118.223479](https://doi.org/10.2967/jnumed.118.223479). | Original article not in the field of interest |
| Bertagna, F.; Albano, D.; Giovanella, L.; Bonacina, M.; Durmo, R.; Giubbini, R.; Treglia, G. 68Ga-PSMA PET Thyroid Incidentalomas. *Hormones (Athens)* **2019**, *18*, 145–149, doi:[10.1007/s42000-019-00106-8](https://doi.org/10.1007/s42000-019-00106-8). | Review not in the field of interest |
| Farolfi, A.; Fendler, W.; Iravani, A.; Haberkorn, U.; Hicks, R.; Herrmann, K.; Walz, J.; Fanti, S. Theranostics for Advanced Prostate Cancer: Current Indications and Future Developments. *Eur Urol Oncol* **2019**, *2*, 152–162, doi:[10.1016/j.euo.2019.01.001](https://doi.org/10.1016/j.euo.2019.01.001). | Review not in the field of interest |
| Piron, S.; De Man, K.; Van Laeken, N.; D’Asseler, Y.; Bacher, K.; Kersemans, K.; Ost, P.; Decaestecker, K.; Deseyne, P.; Fonteyne, V.; et al. Radiation Dosimetry and Biodistribution of 18F-PSMA-11 for PET Imaging of Prostate Cancer. *J Nucl Med* **2019**, *60*, 1736–1742, doi:[10.2967/jnumed.118.225250](https://doi.org/10.2967/jnumed.118.225250). | Original article not in the field of interest |
| Hoberück, S.; Wunderlich, G.; Michler, E.; Hölscher, T.; Walther, M.; Seppelt, D.; Platzek, I.; Zöphel, K.; Kotzerke, J. Dual-Time-Point 64 Cu-PSMA-617-PET/CT in Patients Suffering from Prostate Cancer. *J Labelled Comp Radiopharm* **2019**, *62*, 523–532, doi:[10.1002/jlcr.3745](https://doi.org/10.1002/jlcr.3745). | Original article not in the field of interest |
| Mandiwana, V.; Kalombo, L.; Lemmer, Y.; Labuschagne, P.; Semete-Makokotlela, B.; Sathekge, M.; Ebenhan, T.; Zeevaart, J.R. Preclinical Assessment of 68 Ga-PSMA-617 Entrapped in a Microemulsion Delivery System for Applications in Prostate Cancer PET/CT Imaging. *J Labelled Comp Radiopharm* **2019**, *62*, 332–345, doi:[10.1002/jlcr.3747](https://doi.org/10.1002/jlcr.3747). | Original article not in the field of interest |
| Mai, T.K.; Pham, C.P.; Bui, T.C.; Tran, H.B.; Pham, V.T.; Dao, M.P.; Tran, D.H. Efforts in the Formation and Development of Nuclear Medicine in Vietnam. *Nucl Med Mol Imaging* **2019**, *53*, 83–85, doi:[10.1007/s13139-018-00570-x](https://doi.org/10.1007/s13139-018-00570-x). | Original article not in the field of interest |
| Ferreira, G.; Iravani, A.; Hofman, M.S.; Hicks, R.J. Intra-Individual Comparison of 68Ga-PSMA-11 and 18F-DCFPyL Normal-Organ Biodistribution. *Cancer Imaging* **2019**, *19*, 23, doi:[10.1186/s40644-019-0211-y](https://doi.org/10.1186/s40644-019-0211-y). | Original article not in the field of interest |
| Sathekge, M.; Bruchertseifer, F.; Vorster, M.; Lawal, I.O.; Knoesen, O.; Mahapane, J.; Davis, C.; Reyneke, F.; Maes, A.; Kratochwil, C.; et al. Predictors of Overall and Disease-Free Survival in Metastatic Castration-Resistant Prostate Cancer Patients Receiving 225Ac-PSMA-617 Radioligand Therapy. *J Nucl Med* **2020**, *61*, 62–69, doi:[10.2967/jnumed.119.229229](https://doi.org/10.2967/jnumed.119.229229). | Original article not in the field of interest |
| Calabria, F.; Pichler, R.; Leporace, M.; Wolfsgruber, J.; Coscarelli, P.; Dunzinger, A.; Schillaci, O.; Cascini, G.L.; Bagnato, A. 68Ga/64Cu PSMA Bio-Distribution in Prostate Cancer Patients: Potential Pitfalls for Different Tracers. *Curr Radiopharm* **2019**, *12*, 238–246, doi:[10.2174/1874471012666190515090755](https://doi.org/10.2174/1874471012666190515090755). | Original article not in the field of interest |
| Sahakyan, K.; Li, X.; Lodge, M.A.; Werner, R.A.; Bundschuh, R.A.; Bundschuh, L.; Kulkarni, H.R.; Schuchardt, C.; Baum, R.P.; Pienta, K.J.; et al. Semiquantitative Parameters in PSMA-Targeted PET Imaging with [18F]DCFPyL: Intrapatient and Interpatient Variability of Normal Organ Uptake. *Mol Imaging Biol* **2020**, *22*, 181–189, doi:[10.1007/s11307-019-01376-9](https://doi.org/10.1007/s11307-019-01376-9). | Original article not in the field of interest |
| Guo, H.; Kommidi, H.; Vedvyas, Y.; McCloskey, J.E.; Zhang, W.; Chen, N.; Nurili, F.; Wu, A.P.; Sayman, H.B.; Akin, O.; et al. A Fluorescent, [18F]-Positron-Emitting Agent for Imaging Prostate-Specific Membrane Antigen Allows Genetic Reporting in Adoptively Transferred, Genetically Modified Cells. *ACS Chem Biol* **2019**, *14*, 1449–1459, doi:[10.1021/acschembio.9b00160](https://doi.org/10.1021/acschembio.9b00160). | Original article not in the field of interest |
| Werner, R.A.; Bundschuh, R.A.; Bundschuh, L.; Lapa, C.; Yin, Y.; Javadi, M.S.; Buck, A.K.; Higuchi, T.; Pienta, K.J.; Pomper, M.G.; et al. Semiquantitative Parameters in PSMA-Targeted PET Imaging with [18F]DCFPyL: Impact of Tumor Burden on Normal Organ Uptake. *Mol Imaging Biol* **2020**, *22*, 190–197, doi:[10.1007/s11307-019-01375-w](https://doi.org/10.1007/s11307-019-01375-w). | Original article not in the field of interest |
| Jauw, Y.W.S.; O’Donoghue, J.A.; Zijlstra, J.M.; Hoekstra, O.S.; Menke-van der Houven van Oordt, C.W.; Morschhauser, F.; Carrasquillo, J.A.; Zweegman, S.; Pandit-Taskar, N.; Lammertsma, A.A.; et al. 89Zr-Immuno-PET: Toward a Noninvasive Clinical Tool to Measure Target Engagement of Therapeutic Antibodies In Vivo. *J Nucl Med* **2019**, *60*, 1825–1832, doi:[10.2967/jnumed.118.224568](https://doi.org/10.2967/jnumed.118.224568). | Original article not in the field of interest |
| Sarikaya, I.; Sarikaya, A. Current Status of Radionuclide Renal Cortical Imaging in Pyelonephritis. *J Nucl Med Technol* **2019**, *47*, 309–312, doi:[10.2967/jnmt.119.227942](https://doi.org/10.2967/jnmt.119.227942). | Review not in the field of interest |
| Hohberg, M.; Kobe, C.; Krapf, P.; Täger, P.; Hammes, J.; Dietlein, F.; Zlatopolskiy, B.D.; Endepols, H.; Wild, M.; Neubauer, S.; et al. Biodistribution and Radiation Dosimetry of [18F]-JK-PSMA-7 as a Novel Prostate-Specific Membrane Antigen-Specific Ligand for PET/CT Imaging of Prostate Cancer. *EJNMMI Res* **2019**, *9*, 66, doi:[10.1186/s13550-019-0540-7](https://doi.org/10.1186/s13550-019-0540-7). | Original article not in the field of interest |
| Kumar, N.; Yadav, S.; Kumar, S.; Saurav, K.; Prasad, V.; Vasudeva, P. Comparison of Percentage Free PSA, MRI and GaPSMA PET Scan for Diagnosing Cancer Prostate in Men with PSA between 4 and 20 Ng/Ml. *Indian J Urol* **2019**, *35*, 202–207, doi:[10.4103/iju.IJU_91_19](https://doi.org/10.4103/iju.IJU_91_19). | Original article not in the field of interest |
| Gallyamov, M.; Meyrick, D.; Barley, J.; Lenzo, N. Renal Outcomes of Radioligand Therapy: Experience of 177lutetium-Prostate-Specific Membrane Antigen Ligand Therapy in Metastatic Castrate-Resistant Prostate Cancer. *Clin Kidney J* **2020**, *13*, 1049–1055, doi:[10.1093/ckj/sfz101](https://doi.org/10.1093/ckj/sfz101). | Original article not in the field of interest |
| Green, M.A.; Hutchins, G.D.; Bahler, C.D.; Tann, M.; Mathias, C.J.; Territo, W.; Sims, J.; Polson, H.; Alexoff, D.; Eckelman, W.C.; et al. [68Ga]Ga-P16-093 as a PSMA-Targeted PET Radiopharmaceutical for Detection of Cancer: Initial Evaluation and Comparison with [68Ga]Ga-PSMA-11 in Prostate Cancer Patients Presenting with Biochemical Recurrence. *Mol Imaging Biol* **2020**, *22*, 752–763, doi:[10.1007/s11307-019-01421-7](https://doi.org/10.1007/s11307-019-01421-7). | Original article not in the field of interest |
| Stabile, A.; Muttin, F.; Zamboni, S.; Moschini, M.; Gandaglia, G.; Fossati, N.; Dell’Oglio, P.; Capitanio, U.; Cucchiara, V.; Mazzone, E.; et al. Therapeutic Approaches for Lymph Node Involvement in Prostate, Bladder and Kidney Cancer. *Expert Rev Anticancer Ther* **2019**, *19*, 739–755, doi:[10.1080/14737140.2019.1659135](https://doi.org/10.1080/14737140.2019.1659135). | Review not in the field of interest |
| Kurth, J.; Krause, B.J.; Schwarzenböck, S.M.; Bergner, C.; Hakenberg, O.W.; Heuschkel, M. First-in-Human Dosimetry of Gastrin-Releasing Peptide Receptor Antagonist [177Lu]Lu-RM2: A Radiopharmaceutical for the Treatment of Metastatic Castration-Resistant Prostate Cancer. *Eur J Nucl Med Mol Imaging* **2020**, *47*, 123–135, doi:[10.1007/s00259-019-04504-3](https://doi.org/10.1007/s00259-019-04504-3). | Original article not in the field of interest |
| Mitran, B.; Varasteh, Z.; Abouzayed, A.; Rinne, S.S.; Puuvuori, E.; De Rosa, M.; Larhed, M.; Tolmachev, V.; Orlova, A.; Rosenström, U. Bispecific GRPR-Antagonistic Anti-PSMA/GRPR Heterodimer for PET and SPECT Diagnostic Imaging of Prostate Cancer. *Cancers (Basel)* **2019**, *11*, E1371, doi:[10.3390/cancers11091371](https://doi.org/10.3390/cancers11091371). | Original article not in the field of interest |
| Carlos Dos Santos, J.; Beijer, B.; Bauder-Wüst, U.; Schäfer, M.; Leotta, K.; Eder, M.; Benešová, M.; Kleist, C.; Giesel, F.; Kratochwil, C.; et al. Development of Novel PSMA Ligands for Imaging and Therapy with Copper Isotopes. *J Nucl Med* **2020**, *61*, 70–79, doi:[10.2967/jnumed.119.229054](https://doi.org/10.2967/jnumed.119.229054). | Original article not in the field of interest |
| Evangelista, L.; Zattoni, F.; Alongi, P. 68Ga-Dotatoc vs. 18F-FDG vs. Radiolabelled PSMA PET/CT in Renal Cancer Patients. *Ann Transl Med* **2019**, *7*, S150, doi:[10.21037/atm.2019.06.28](https://doi.org/10.21037/atm.2019.06.28). | Editorial in the field of interest |
| Van de Wiele, C.; Sathekge, M.; de Spiegeleer, B.; de Jonghe, P.J.; Beels, L.; Maes, A. PSMA-Targeting Positron Emission Agents for Imaging Solid Tumors Other Than Non-Prostate Carcinoma: A Systematic Review. *Int J Mol Sci* **2019**, *20*, E4886, doi:[10.3390/ijms20194886](https://doi.org/10.3390/ijms20194886). | Review in the field of interest |
| Pozzessere, C.; Bassanelli, M.; Ceribelli, A.; Rasul, S.; Li, S.; Prior, J.O.; Cicone, F. Renal Cell Carcinoma: The Oncologist Asks, Can PSMA PET/CT Answer? *Curr Urol Rep* **2019**, *20*, 68, doi:[10.1007/s11934-019-0938-9](https://doi.org/10.1007/s11934-019-0938-9). | Review in the field of interest |
| Dietlein, F.; Kobe, C.; Hohberg, M.; Zlatopolskiy, B.D.; Krapf, P.; Endepols, H.; Täger, P.; Hammes, J.; Heidenreich, A.; Persigehl, T.; et al. Intraindividual Comparison of 18F-PSMA-1007 with Renally Excreted PSMA Ligands for PSMA PET Imaging in Patients with Relapsed Prostate Cancer. *J Nucl Med* **2020**, *61*, 729–734, doi:[10.2967/jnumed.119.234898](https://doi.org/10.2967/jnumed.119.234898). | Original article not in the field of interest |
| A, A.; A, F.; E, P.; G, S.; R, S. Asymptomatic Metastasis to Thyroid Cartilage Detected by 18F-Choline and 64Cu-PSMA PET/CT as a Single Site of Disease Relapse in a Patient With Castration-Resistant Prostate Carcinoma. *Clinical nuclear medicine* **2020**, *45*, doi:[10.1097/RLU.0000000000002786](https://doi.org/10.1097/RLU.0000000000002786). | Case report in the field of interest |
| Michalski, K.; Mix, M.; Meyer, P.T.; Ruf, J. Determination of Whole-Body Tumour Burden on [68Ga]PSMA-11 PET/CT for Response Assessment of [177Lu]PSMA-617 Radioligand Therapy: A Retrospective Analysis of Serum PSA Level and Imaging Derived Parameters before and after Two Cycles of Therapy. *Nuklearmedizin* **2019**, *58*, 443–450, doi:[10.1055/a-1035-9052](https://doi.org/10.1055/a-1035-9052). | Original article not in the field of interest |
| Zhang, F.; Fu, Y.; Chen, M.; Wang, F.; Qiu, X.; Guo, H. Renal Pelvis Metastasis From Prostatic Adenocarcinoma Missed by PSMA PET: A Case Report. *Urology* **2020**, *136*, e3–e4, doi:[10.1016/j.urology.2019.11.005](https://doi.org/10.1016/j.urology.2019.11.005). | Case report not in the field of interest |
| ùVornov, J.J.; Peters, D.; Nedelcovych, M.; Hollinger, K.; Rais, R.; Slusher, B.S. Looking for Drugs in All the Wrong Places: Use of GCPII Inhibitors Outside the Brain. *Neurochem Res* **2020**, *45*, 1256–1267, doi:[10.1007/s11064-019-02909-y](https://doi.org/10.1007/s11064-019-02909-y). | Review not in the field of interest |
| Baiocco, S.; Matteucci, F.; Mezzenga, E.; Caroli, P.; Di Iorio, V.; Cittanti, C.; Bevilacqua, A.; Paganelli, G.; Sarnelli, A. SUV95th as a Reliable Alternative to SUVmax for Determining Renal Uptake in [68Ga] PSMA PET/CT. *Mol Imaging Biol* **2020**, *22*, 1070–1077, doi:[10.1007/s11307-019-01451-1](https://doi.org/10.1007/s11307-019-01451-1). | Original article not in the field of interest |
| Oh, S.W.; Wurzer, A.; Teoh, E.J.; Oh, S.; Langbein, T.; Krönke, M.; Herz, M.; Kropf, S.; Wester, H.-J.; Weber, W.A.; et al. Quantitative and Qualitative Analyses of Biodistribution and PET Image Quality of a Novel Radiohybrid PSMA, 18F-RhPSMA-7, in Patients with Prostate Cancer. *J Nucl Med* **2020**, *61*, 702–709, doi:[10.2967/jnumed.119.234609](https://doi.org/10.2967/jnumed.119.234609). | Original article not in the field of interest |
| Begum, N.J.; Glatting, G.; Wester, H.-J.; Eiber, M.; Beer, A.J.; Kletting, P. The Effect of Ligand Amount, Affinity and Internalization on PSMA-Targeted Imaging and Therapy: A Simulation Study Using a PBPK Model. *Sci Rep* **2019**, *9*, 20041, doi:[10.1038/s41598-019-56603-8](https://doi.org/10.1038/s41598-019-56603-8). | Original article not in the field of interest |
| Rosar, F.; Dewes, S.; Ries, M.; Schaefer, A.; Khreish, F.; Maus, S.; Bohnenberger, H.; Linxweiler, J.; Bartholomä, M.; Ohlmann, C.; et al. New Insights in the Paradigm of Upregulation of Tumoral PSMA Expression by Androgen Receptor Blockade: Enzalutamide Induces PSMA Upregulation in Castration-Resistant Prostate Cancer Even in Patients Having Previously Progressed on Enzalutamide. *Eur J Nucl Med Mol Imaging* **2020**, *47*, 687–694, doi:[10.1007/s00259-019-04674-0](https://doi.org/10.1007/s00259-019-04674-0). | Original article not in the field of interest |
| Mittlmeier, L.M.; Unterrainer, M.; Todica, A.; Cyran, C.C.; Rodler, S.; Bartenstein, P.; Stief, C.G.; Ilhan, H.; Staehler, M. PSMA PET/CT for Tyrosine-Kinase Inhibitor Monitoring in Metastatic Renal Cell Carcinoma. *Eur J Nucl Med Mol Imaging* **2020**, *47*, 2216–2217, doi:[10.1007/s00259-019-04636-6](https://doi.org/10.1007/s00259-019-04636-6). | Case report in the field of interest |
| Siva, S.; Udovicich, C.; Tran, B.; Zargar, H.; Murphy, D.G.; Hofman, M.S. Expanding the Role of Small-Molecule PSMA Ligands beyond PET Staging of Prostate Cancer. *Nat Rev Urol* **2020**, *17*, 107–118, doi:[10.1038/s41585-019-0272-5](https://doi.org/10.1038/s41585-019-0272-5). | Review in the field of interest |
| Li, L.; Jaraquemada-Peláez, M. de G.; Aluicio-Sarduy, E.; Wang, X.; Jiang, D.; Sakheie, M.; Kuo, H.-T.; Barnhart, T.E.; Cai, W.; Radchenko, V.; et al. [Nat/44Sc(Pypa)]-: Thermodynamic Stability, Radiolabeling, and Biodistribution of a Prostate-Specific-Membrane-Antigen-Targeting Conjugate. *Inorg Chem* **2020**, *59*, 1985–1995, doi:[10.1021/acs.inorgchem.9b03347](https://doi.org/10.1021/acs.inorgchem.9b03347). | Original article not in the field of interest |
| Qureshi, P.A.A.A.; Asghar, N.; Bashir, H.; Niazi, I.K.; Akhtar, N. The Hot Pleura: Isolated Pleural Metastases From Renal Cell Carcinoma. *Clin Nucl Med* **2020**, *45*, 211–213, doi:[10.1097/RLU.0000000000002909](https://doi.org/10.1097/RLU.0000000000002909). | Case report not in the field of interest |
| Potemkin, R.; Strauch, B.; Kuwert, T.; Prante, O.; Maschauer, S. Development of 18F-Fluoroglycosylated PSMA-Ligands with Improved Renal Clearance Behavior. *Mol Pharm* **2020**, *17*, 933–943, doi:[10.1021/acs.molpharmaceut.9b01179](https://doi.org/10.1021/acs.molpharmaceut.9b01179). | Original article not in the field of interest |
| Sharan, B.; Chiliveru, S.; Bagga, J.; Kohli, S.; Bharadwaj, A.; Vaid, A.K.; Kumar, C. Substantial Tumor Regression in Prostate Cancer Patient with Extensive Skeletal Metastases upon Immunotherapy (APCEDEN): A Case Report. *Medicine (Baltimore)* **2020**, *99*, e18889, doi:[10.1097/MD.0000000000018889](https://doi.org/10.1097/MD.0000000000018889). | Case report not in the field of interest |
| Kurash, M.M.; Gill, R.; Khairulin, M.; Harbosh, H.; Keidar, Z. 68Ga-Labeled PSMA-11 (68Ga-IsoPROtrace-11) Synthesized with Ready to Use Kit: Normal Biodistribution and Uptake Characteristics of Tumour Lesions. *Sci Rep* **2020**, *10*, 3109, doi:[10.1038/s41598-020-60099-y](https://doi.org/10.1038/s41598-020-60099-y). | Original article not in the field of interest |
| Mittlmeier, L.M.; Unterrainer, M.; Todica, A.; Clevert, D.A.; Cyran, C.C.; Schmoeckel, E.; Rodler, S.; Bartenstein, P.; Stief, C.G.; Ilhan, H.; et al. Advanced Molecular Imaging in Histologically Verified Metanephric Adenoma. *Urology* **2020**, *140*, e10–e11, doi:[10.1016/j.urology.2020.02.025](https://doi.org/10.1016/j.urology.2020.02.025). | Case report not in the field of interest |
| Marafi, F.; Sasikumar, A.; Al-Terki, A.; Alfeeli, M. 18F-PSMA 1007 in Suspected Renal Cell Carcinoma. *Clin Nucl Med* **2020**, *45*, 377–378, doi:[10.1097/RLU.0000000000003002](https://doi.org/10.1097/RLU.0000000000003002). | Case report in the field of interest |
| Mosayebnia, M.; Hajimahdi, Z.; Beiki, D.; Rezaeianpour, M.; Hajiramezanali, M.; Geramifar, P.; Sabzevari, O.; Amini, M.; Hatamabadi, D.; Shahhosseini, S. Design, Synthesis, Radiolabeling and Biological Evaluation of New Urea-Based Peptides Targeting Prostate Specific Membrane Antigen. *Bioorg Chem* **2020**, *99*, 103743, doi:[10.1016/j.bioorg.2020.103743](https://doi.org/10.1016/j.bioorg.2020.103743). | Original article not in the field of interest |
| Kelly, J.M.; Ponnala, S.; Amor-Coarasa, A.; Zia, N.A.; Nikolopoulou, A.; Williams, C.; Schlyer, D.J.; DiMagno, S.G.; Donnelly, P.S.; Babich, J.W. Preclinical Evaluation of a High-Affinity Sarcophagine-Containing PSMA Ligand for 64Cu/67Cu-Based Theranostics in Prostate Cancer. *Mol Pharm* **2020**, *17*, 1954–1962, doi:[10.1021/acs.molpharmaceut.0c00060](https://doi.org/10.1021/acs.molpharmaceut.0c00060). | Original article not in the field of interest |
| Kamaldeep; Wanage, G.; Sahu, S.K.; Maletha, P.; Adnan, A.; Suman, S.; Basu, S.; Das, T.; Banerjee, S. Examining Absorbed Doses of Indigenously Developed 177Lu-PSMA-617 in Metastatic Castration-Resistant Prostate Cancer Patients at Baseline and During Course of Peptide Receptor Radioligand Therapy. *Cancer Biother Radiopharm* **2021**, *36*, 292–304, doi:[10.1089/cbr.2020.3640](https://doi.org/10.1089/cbr.2020.3640). | Original article not in the field of interest |
| Harsini, S.; Saprunoff, H.; Alden, T.; Mohammadi, B.; Wilson, D.; Bénard, F. The Effects of Monosodium Glutamate on PSMA Radiotracer Uptake in Men with Recurrent Prostate Cancer: A Prospective, Randomized, Double-Blind, Placebo-Controlled Intraindividual Imaging Study. *J Nucl Med* **2021**, *62*, 81–87, doi:[10.2967/jnumed.120.246983](https://doi.org/10.2967/jnumed.120.246983). | Original article not in the field of interest |
| Uprimny, C.; Bayerschmidt, S.; Kroiss, A.S.; Fritz, J.; Nilica, B.; Svirydenka, A.; Decristoforo, C.; di Santo, G.; von Guggenberg, E.; Horninger, W.; et al. Impact of Forced Diuresis with Furosemide and Hydration on the Halo Artefact and Intensity of Tracer Accumulation in the Urinary Bladder and Kidneys on [68Ga]Ga-PSMA-11-PET/CT in the Evaluation of Prostate Cancer Patients. *Eur J Nucl Med Mol Imaging* **2021**, *48*, 123–133, doi:[10.1007/s00259-020-04846-3](https://doi.org/10.1007/s00259-020-04846-3). | Original article not in the field of interest |
| Ried, K.; Tamanna, T.; Matthews, S.; Eng, P.; Sali, A. New Screening Test Improves Detection of Prostate Cancer Using Circulating Tumor Cells and Prostate-Specific Markers. *Front Oncol* **2020**, *10*, 582, doi:[10.3389/fonc.2020.00582](https://doi.org/10.3389/fonc.2020.00582). | Original article not in the field of interest |
| Chen, E.-J.; Tan, T.H.; Chew, M.T.; Chye, P.C. 68Ga-PSMA PET/CT and 18F-FDG PET/CT in Renal Cell Carcinoma. *Clin Nucl Med* **2020**, *45*, e317–e319, doi:[10.1097/RLU.0000000000003053](https://doi.org/10.1097/RLU.0000000000003053). | Case report in the field of interest |
| Lee, I.; Lim, I.; Byun, B.H.; Kim, B.I.; Choi, C.W.; Woo, S.-K.; Lee, K.C.; Kang, J.H.; Kil, H.S.; Park, C.; et al. A Microdose Clinical Trial to Evaluate [18F]Florastamin as a Positron Emission Tomography Imaging Agent in Patients with Prostate Cancer. *Eur J Nucl Med Mol Imaging* **2021**, *48*, 95–102, doi:[10.1007/s00259-020-04883-y](https://doi.org/10.1007/s00259-020-04883-y). | Original article not in the field of interest |
| Barna, S.; Haug, A.R.; Hartenbach, M.; Rasul, S.; Grubmüller, B.; Kramer, G.; Blaickner, M. Dose Calculations and Dose-Effect Relationships in 177Lu-PSMA I&T Radionuclide Therapy for Metastatic Castration-Resistant Prostate Cancer. *Clin Nucl Med* **2020**, *45*, 661–667, doi:[10.1097/RLU.0000000000003157](https://doi.org/10.1097/RLU.0000000000003157). | Original article not in the field of interest |
| Farolfi, A.; Koschel, S.; Murphy, D.G.; Fanti, S. PET Imaging in Urology: A Rapidly Growing Successful Collaboration. *Curr Opin Urol* **2020**, *30*, 623–627, doi:[10.1097/MOU.0000000000000800](https://doi.org/10.1097/MOU.0000000000000800). | Review in the field of interest |
| Chahinian, R.; El-Amine, A.; Matar, S.; Annan, M.; Shamseddine, A.; Haidar, M. 68Ga-Prostate-Specific Membrane Antigen, A Potential Radiopharmaceutical in PET/CT To Detect Primary Cholangiocarcinoma. *Asia Ocean J Nucl Med Biol* **2020**, *8*, 136–140, doi:[10.22038/AOJNMB.2020.46939.1314](https://doi.org/10.22038/AOJNMB.2020.46939.1314). | Case report not in the field of interest |
| Khansa, Z.; Neaimeh, N.; Korek, M.; Haidar, M. Can SUVmax of 68Ga-Labeled PSMA Ligand and 18F-Choline PET/CT Be Used to Predict the Radiation Dose in Prostate Cancer Patients? *Health Phys* **2021**, *120*, 80–85, doi:[10.1097/HP.0000000000001287](https://doi.org/10.1097/HP.0000000000001287). | Original article not in the field of interest |
| Bendre, S.; Zhang, Z.; Kuo, H.-T.; Rousseau, J.; Zhang, C.; Merkens, H.; Roxin, Á.; Bénard, F.; Lin, K.-S. Evaluation of Met-Val-Lys as a Renal Brush Border Enzyme-Cleavable Linker to Reduce Kidney Uptake of 68Ga-Labeled DOTA-Conjugated Peptides and Peptidomimetics. *Molecules* **2020**, *25*, E3854, doi:[10.3390/molecules25173854](https://doi.org/10.3390/molecules25173854). | Original article not in the field of interest |
| Kuo, H.-T.; Lin, K.-S.; Zhang, Z.; Uribe, C.F.; Merkens, H.; Zhang, C.; Bénard, F. 177Lu-Labeled Albumin-Binder-Conjugated PSMA-Targeting Agents with Extremely High Tumor Uptake and Enhanced Tumor-to-Kidney Absorbed Dose Ratio. *J Nucl Med* **2021**, *62*, 521–527, doi:[10.2967/jnumed.120.250738](https://doi.org/10.2967/jnumed.120.250738). | Original article not in the field of interest |
| Lamb, J.; Fischer, E.; Rosillo-Lopez, M.; Salzmann, C.G.; Holland, J.P. Multi-Functionalised Graphene Nanoflakes as Tumour-Targeting Theranostic Drug-Delivery Vehicles. *Chem Sci* **2019**, *10*, 8880–8888, doi:[10.1039/c9sc03736e](https://doi.org/10.1039/c9sc03736e). | Original article not in the field of interest |
| Sarikaya, I.; Alqallaf, A.; Sarikaya, A. Renal Cortical 68Ga-PSMA-11 PET and 99mTc-DMSA Images. *J Nucl Med Technol* **2021**, *49*, 30–33, doi:[10.2967/jnmt.120.248922](https://doi.org/10.2967/jnmt.120.248922). | Original article not in the field of interest |
| V, K.; R, F.; W, L.; Ld, J.-F.; C, S.-R.; E, E.; M, C.; M, M.; M, B.; Ca, U.; et al. Biodistribution and Dosimetry of a Single Dose of Albumin-Binding Ligand [177Lu]Lu-PSMA-ALB-56 in Patients with MCRPC. *European journal of nuclear medicine and molecular imaging* **2021**, *48*, doi:[10.1007/s00259-020-05022-3](https://doi.org/10.1007/s00259-020-05022-3). | Original article not in the field of interest |
